# High-throughput, low volume d-ROMs and BAP assays: 384-well plate method for large-scale studies

**DOI:** 10.1265/ehpm.25-00354

**Published:** 2026-07-03

**Authors:** Goki Yamada, Sudarma Bogahawaththa, Yuichiro Nishida, Mikako Horita, Kazuhiro Kawamoto, Yasuyuki Maeda, Mikiko Tokiya, Megumi Hara, Tadayuki Tsujita, Akiko Matsumoto

**Affiliations:** 1 Department of Preventive Medicine, Faculty of Medicine, Saga University, Nabeshima, Saga 849-8501, Japan; 2 The United Graduate School of Agricultural Sciences, Kagoshima University, Korimoto, Kagoshima 890-0065, Japan; 3 Laboratory of Biochemistry, Faculty of Agriculture, Saga University, Honjo, Saga 840-8502, Japan; 4 Department of Social and Environmental Medicine, Faculty of Medicine, Saga University, Nabeshima, Saga 849-8501, Japan

**Keywords:** Oxidative balance, d-ROMs, BAP, High-throughput, Pipetting robot, Validation

## Abstract

**Background:**

The d-ROMs and BAP serum tests are promising markers of oxidative balance in research settings. Conventional assay systems require 30 µL of serum and approximately 10 minutes to analyze a single sample. Although these systems have the advantages of calibration-free operation and the flexibility to perform individual tests at any time, their limited throughput and relatively high cost restrict their use in large-scale studies.

**Methods:**

To address this issue, we developed a small-scale, semi-automated measurement system incorporating a pipetting robot and a 384-well plate. The system requires only 5.0 µL of serum sample and can process up to 144 samples simultaneously for both d-ROMs and BAP assays (a total of 288 measurements).

**Results:**

Based on nonlinearity testing, 450 nm was selected as the optimal wavelength. To maintain linearity across various sample types, including turbid serum, d-ROMs required incubation at 4 °C for one week, and BAP required centrifugation to remove precipitates. Intra- and inter-assay, as well as inter-day, coefficients of variation ranged from 1% to 9% for d-ROMs and 2% to 17% for BAP. Inter-operator variations were <9%. Lipemic serum showed abnormally low BAP values using the conventional method but normal values using our method. In non-turbid samples, the current and conventional methods showed high agreement. Deming regression analysis of log_10_-transformed d-ROMs values yielded a slope of 1.06 (95% confidence interval between 0.99–1.13), and an intercept of −0.12 (−0.28 to 0.04). The same analysis for BAP yielded a slope of 0.97 (0.821–1.13) and an intercept of −0.12 (−0.391 to 0.625). Stability testing indicated that serum should be stored at −80 °C in volumes ≥100 µL for standard 1.5 mL tubes or ≥21 µL for 96-well format tubes. Our method reduced serum requirements by 83%, reagent use by 93% for d-ROMs and 86% for BAP. Although colorimetric measurement is required after one week of incubation, actual labor time was reduced by approximately 80%.

**Conclusions:**

We have developed a high-throughput, cost-effective in-house assay system for large-scale studies. This system provides accurate measurements of oxidative stress markers even in turbid samples, facilitating robust evidence-based research on oxidative stress and health.

**Supplementary information:**

The online version contains supplementary material available at https://doi.org/10.1265/ehpm.25-00354.

## 1. Introduction

Oxidative stress is defined as an imbalance between the production of reactive oxygen species (ROS) and the efficiency of the antioxidative defense system [1–3]. Indicators of oxidative stress levels and antioxidative capacity have been proposed as critical biomarkers linked to chronic diseases, aging, and lifestyle factors [4–6]. However, they have not yet been used in large-scale studies; therefore, there is a growing demand to assess these parameters across thousands of individuals efficiently. Various biomarkers are used to assess ROS production, including thiobarbituric acid reactive substances, serum oxidized low-density lipoprotein, and urinary 8-hydroxy-2′-deoxyguanosine [7–9]. On the other hand, antioxidative potential is measured by indicators such as superoxide dismutase activity, catalase activity, blood glutathione concentration, and paraoxonase activity [10, 11]. However, detecting these oxidative stress markers typically requires separate samples and specialized testing equipment for each, making it unsuitable for large-scale, high-throughput studies.

Recently, the diacron-reactive oxygen metabolites (d-ROMs) and biological antioxidant potential (BAP) test has gained attention as a method capable of simultaneously measuring both ROS levels and antioxidative potential using the same sample and testing equipment [12, 13]. The d-ROMs test measures hydroperoxides (ROOH) of lipids, proteins, amino acids, nucleic acids, and other biomolecules that have been oxidized by ROS, using a colorimetric reaction [14]. The BAP test, on the other hand, evaluates the total reducing potential in the body by measuring the ability of antioxidants in the blood to reduce ferric ions (Fe^3+^) to ferrous ions (Fe^2+^), also using a colorimetric reaction [15].

The conventional d-ROMs/BAP methodology requires a dedicated device equipped with a 37 °C incubator and a spectrophotometer at 505 nm wavelength. This system is designed for calibration-free operation and requires a dedicated reagent kit, a total of 30 µL of serum per sample, and approximately 10 minutes of processing time [16]. The cost per sample, including labor costs, is relatively high, at over 3,000 yen. Although the ability to perform individual assays on demand represents a practical advantage of this system, its scalability for high-throughput applications remains limited. For this reason, these tests have not been applied to large-scale studies, and their preventive and clinical value has not been fully verified.

Accordingly, there is a clear and pressing need to develop analytical approaches that reduce sample consumption, shorten processing time, and lower overall costs, while preserving the analytical reliability of the d-ROMs and BAP assays. As one practical strategy to achieve these goals, we established a semi-automated system capable of simultaneously analyzing up to 144 samples, thereby enabling efficient, rapid, and cost-effective measurement of d-ROMs and BAP without compromising assay performance. This could accelerate the accumulation of evidence, clarify preventive and clinical implications, and potentially establish d-ROMs and BAP as common clinical tests.

## 2. Materials and methods

### 2.1 Preparation of standard serum for calibration

Whole blood from 12 healthy volunteers was collected and left at room temperature for 40 minutes. The serum was then obtained via centrifugation at 1,200 × g at 4 °C for 15 minutes and pooled into a single tube (approx. 90 mL). d-ROMs and BAP levels were measured using the conventional redox analyzer: FREE Carrio Duo system (Wismerll, Tokyo, Japan); a dedicated analyzer. The remaining sample aliquots were stored at −80 °C for subsequent analysis.

### 2.2 Sample preparation and measurement procedure

The analytical procedure was illustrated in detail in Fig. S1. Figure S1A shows that standard serum was diluted to six different concentrations corresponding to 115 to 575 U.CARR (Carratelli Units, equivalent to 0.08 mg H_2_O_2_/dL, https://www.wismerll.co.jp/reagent/d-roms/index.html) for d-ROMs and 1,082 to 5,410 µM for BAP, respectively (STD1 to 6). Unknown serum samples were diluted 1:4 with saline prior to analysis. Almost all liquid-handling steps, including dispensing and mixing of reaction reagents and transfer of the BAP reaction supernatant, were performed using an automated pipetting robot, Andrew+ (Waters, Milford, MA, USA), to ensure consistency and reproducibility. The protocol includes two major methodological modifications from the conventional system. First, a supernatant extraction step was added for BAP as shown in Figs. S1F to J. After mixing the BAP reagent and serum in 96-well plates (BAP extraction plates, Fig. S1G), the plates were centrifuged (4,000 × g at 4 °C for 5 minutes) to remove the precipitates (Fig. S1I). The resulting supernatant, excluding any precipitate, was transferred to the other half of the 384-well plate (aspiration strategy is detailed in Fig. S1J). Second, a two-step color measurement was implemented; BAP was measured after a 10-minute incubation at 37 °C (Figs. S1L and M), and then d-ROMs measurement after a seven-day incubation at 4 °C (Figs. S1N and O). During the seven-day incubation period, the plates were sealed with a pressure-sensitive film (MicroAmp™ Optical Adhesive Film, Applied Biosystems, Foster City, CA, USA) to prevent evaporation and were stored in a light-protected environment. Then, the absorbance at the optimized wavelength was measured using a plate reader (Multiskan FC, Thermo Fisher Scientific, Waltham, MA, USA).

### 2.3. Calibration procedure

**Fig. 1:**
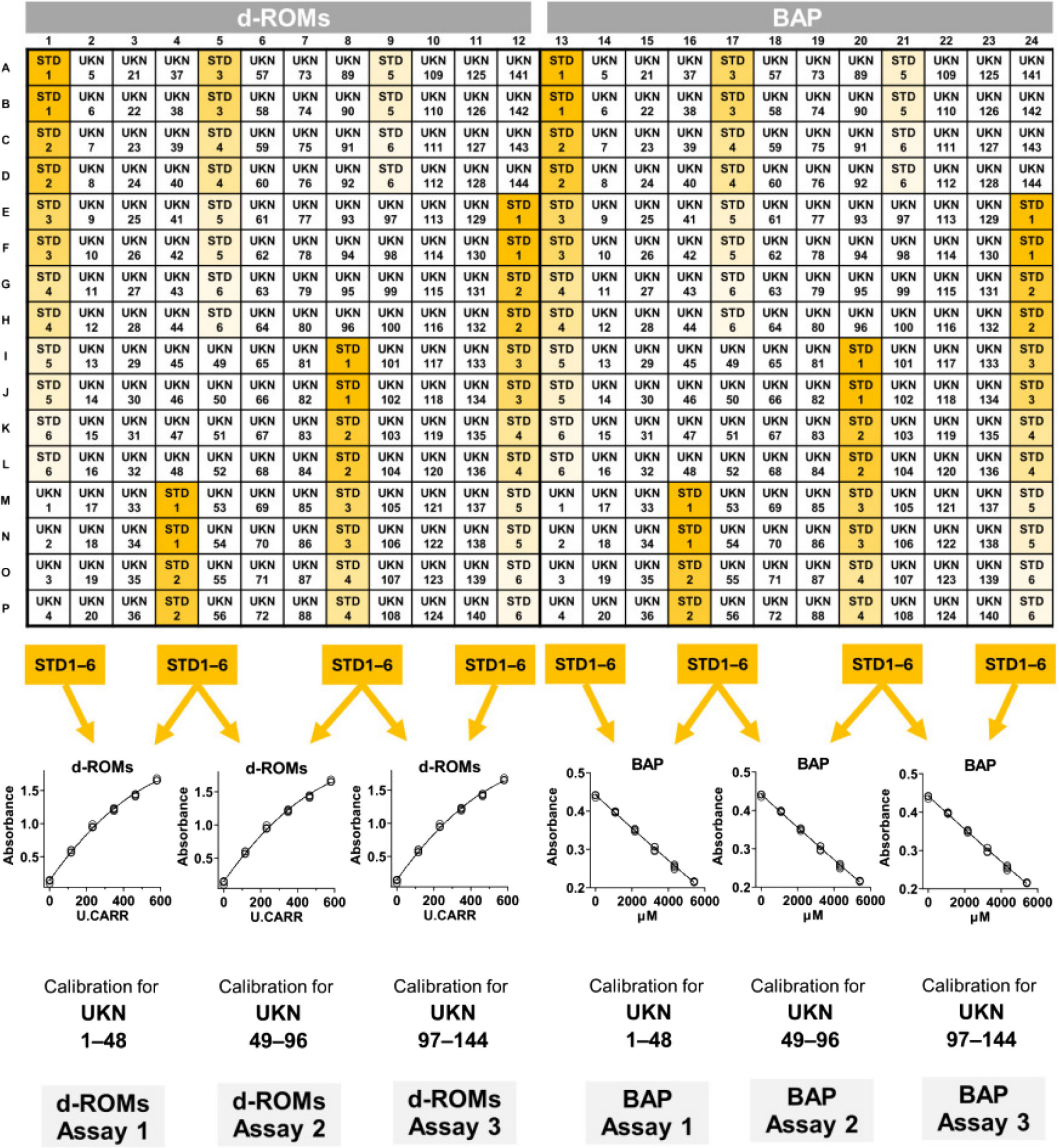
Plate layout and calibration strategy for small-scale d-ROMs and BAP assays. The plate is divided with d-ROMs on the left and BAP on the right. Each section contains three assay sections. Each assay section contains groups of 48 unknown samples (UKN), which are flanked by six-point standard series (STD1–6). A localized calibration curve is generated for each group of unknowns by averaging the two adjacent standard series to minimize the effect of column position. The final concentrations are calibrated using these localized curves.

As shown in Fig. 1, each 384-well plate was divided into three assays (assays 1–3) for each of d-ROMs and BAP to minimize the deviation in color reaction due to the time difference caused by the dispensing process. A single assay consists of 72 measurements: STD1 to 6 in duplicate (e.g., wells 1A-1L in assay 1 for d-ROMs), 48 unknown samples (e.g., wells 1M–4L in assay 1 for d-ROMs), and an additional series of standards (e.g., wells 4M-4P and 5A-5H in assay 1 for d-ROMs). Consequently, one standard curve is generated using 24 standard samples, and the concentration of each unknown sample is calculated based on this curve.

### 2.4 Linearity, recovery and intra-plate variation

Dilution linearity was examined using serum samples at 450, 492, and 560 nm wavelengths, modeled in quadruplicate across the clinical range, with linearity and second-order polynomial regression compared through non-linear tests. The recovery rate was determined by analyzing a five-level serum dilution series and calculating the ratio of measured values to expected values based on conventional method results. For intra-plate variation, a single serum sample was dispensed across all 192 wells for each assay (d-ROMs and BAP) to visualize absorbance distribution and potential row or column effect.

### 2.5 Quality control tracking

STD2 and STD4 were tracked for quality control purposes. Within each 384-well plate, the d-ROMs and BAP values of STD2 and STD4 were calculated using standard curves from adjacent assays to monitor the stability of the calibration curves. STD2 and STD4 in columns 1, 4, 5, 13, 16, and 17 were calculated using adjacent assays on the right (assays 2 and 3), and the rest referred to the one on the left (assays 1 and 2). Since our assay is designed to quantify unknown samples flanked by a pair of 12 standards (a total of 24 standards), this calculation generates lower and higher values than the original values by using the calibration curves from the adjacent right and left assays, respectively (i.e., use of unapplicable standard curves). To more sensitively monitor within-plate stability, we used this method for 27 discontinuous days and produced an $\bar{X}$ –R chart.

### 2.6 Reproducibility test

To verify the reproducibility, we performed repeated measurements of normal serum A–D, lipemic serum E–F, and hemolyzed serum G–H. The d-ROMs and BAP measurements were initially performed on fresh samples using the conventional method. Measurements of frozen serum A and B, C and D, and E–H were then repeated 132 times, 36 times, and 12 times, respectively using the current method.

To evaluate Inter-operator variations, three operators each diluted three different serum samples by mixing 5.0 µL of serum with 20 µL of saline. The resulting serum samples were subjected to d-ROMs and BAP measurements using a pipetting robot in quadruplicate.

### 2.7 Inter-method agreement

d-ROMs and BAP levels were obtained for 11 serum samples without obvious turbidity using both the conventional method and the current method. Agreement between the two methods was evaluated via Deming regression and Bland-Altman plots using log-transformed data.

### 2.8 Sample stability test

To evaluate the effects of freeze-thaw cycles on serum stability, samples from healthy volunteers were stored in two types of containers: standard 1.5 mL tubes and 96-format 0.7 mL tubes (Fig. S2). For the 1.5 mL tubes, seven serum samples were prepared at volumes of 10 and 100 µL and subjected to one to five freeze-thaw cycles at −80 °C. For the 0.7 mL tubes, four samples were prepared at 21, 70, and 210 µL and stored at either −80 °C or −20 °C, undergoing one, four, or eight cycles. Each cycle consisted of thawing, a 3-hour incubation at 25 °C, and refreezing.

### 2.9 Statistical analysis

Statistical analyses were performed using SAS for Windows version 9.4 (SAS Institute, Cary, NC, USA) and Prism 9 (GraphPad Software, Boston, MA, USA). P values less than 0.05 were considered significant. To assess the nonlinearity of the calibration curves, we compared a simple linear regression model with a quadratic model using the extra sum-of-squares F test. Inter-method agreement was analyzed using Deming regression to estimate the constant and proportional bias. Additionally, a Bland-Altman analysis was used to calculate the mean bias and the 95% limits of agreement (mean bias ± 1.96 SD). The effect of freeze-thaw cycle was estimated using mixed-effect models that accounted for the random effect by serum specificity to account for inherent variability (SAS code is provided in the supplementary materials).

## 3. Results

### 3.1 Optimization for dilution linearity

**Fig. 2:**
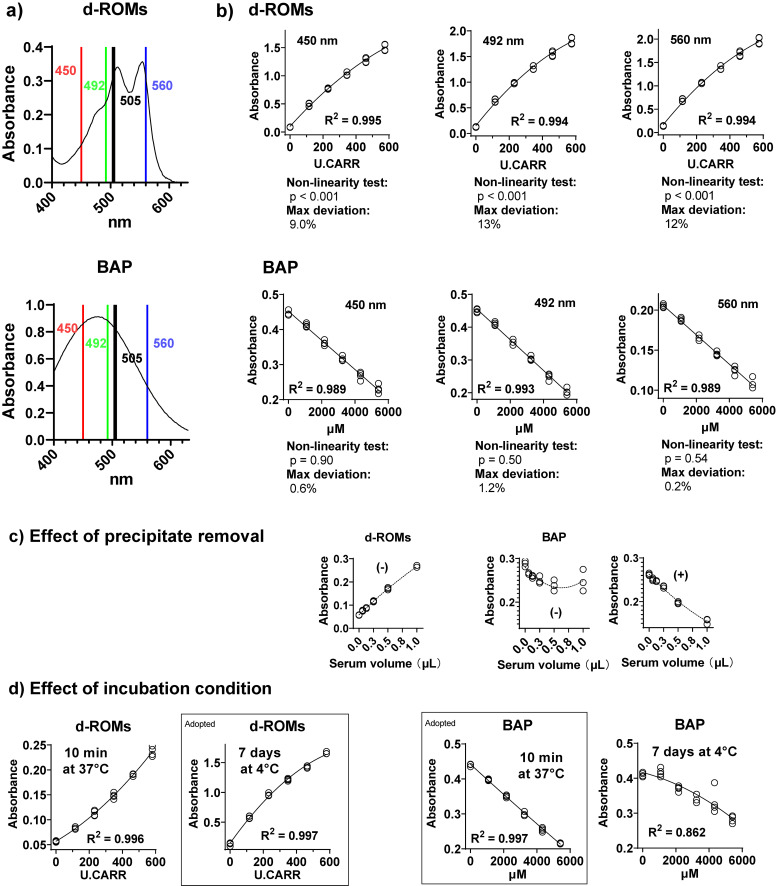
Dilution linearity in small-scale d-ROMs and BAP measurement. a) Absorbance spectra of serum after coloring: The vertical bold and black lines in the spectra indicate the wavelength adopted in the conventional method (505 nm), and the colored lines indicate the candidate wavelengths in the current study (450, 492, and 560 nm). b) Dilution linearity: Calibration curves covering the clinical range were modeled in quadruplicate. R^2^, the coefficient of determination. Non-linear tests were performed to compare linear and second-order polynomial regression. Maximum deviation from the linear model at non-zero levels is also presented. c) Effect of precipitate removal on dilution linearity: Absorbance measurements were taken for dilution series at 480 nm after a 10-minute incubation at 37 °C, both without (−) or with (+) centrifugation at 4,000 × g at 4 °C for 5 minutes to remove precipitate to improve linearity. d) Effect of incubation conditions on dilution linearity: Absorbance measurements at 450 nm were taken after a 10-minute incubation at 37 °C or after an additional seven-day incubation at 4 °C. R^2^ values are for second-order polynomial regression.

As shown in Fig. 2a, d-ROMs and BAP exhibit an absorbance peak between 400 and 600 nm; conventional methods utilize an absorbance of 505 nm. However, since this wavelength is inaccessible at our facility, we searched for an alternative. Although dilution linearity was confirmed between 450 and 560 nm, d-ROMs showed a better fit with quadratic regression equations. Color measurement at 450 nm showed the least deviation from the linear regression equation (Fig. 2b). In the BAP assay, no difference was detected between the linear regression and quadratic curve fits at any of the tested wavelengths. Consequently, we adopted 450 nm as the measurement wavelength.

As shown in Fig. 2c, certain sera formed precipitates upon mixing with the BAP reagent, interfering with the dilution linearity. To address this issue, we introduced an additional centrifugation step to extract the supernatant from the mixture (a step not included in conventional systems). The aspiration was performed at a slow speed with the tip position set relative to the liquid surface to ensure the pellet remained undisturbed. With this modification, lipemic serum, which is considered an inappropriate sample for conventional BAP methods, showed normal values comparable values to those of the general Japanese population [17, 18]: abnormally low values, 978 and 758 µM using the conventional system, and 1,843 and 1,751 µM using the current method (Table S1).

As shown in Table S2, the 7-day incubation at 4 °C demonstrated a lower inter-day CV (3–5%) for d-ROMs STD1–4 (230–575 U.CARR) compared to the 10-minute incubation at 37 °C (7–10%). Although the CV values for STD5 and 6 (0 and 115 U.CARR) were higher (7 and 20%), we adopted the seven-day incubation at 4 °C to prioritize higher d-ROMs. This strategy provides greater accuracy in concentration ranges more directly linked to disease risk. Furthermore, Table S1 shows that a short incubation time produced significantly higher values for lipemic samples, serum E and F, 712 and 536 U.CARR, representing 188% and 215% of the values obtained using the conventional system, respectively. However, with a seven-day incubation period, the current method yielded closer values of 111% and 116%, respectively (Table S1). As the BAP test showed disrupted dilution linearity after a seven-day incubation, we decided to perform color measurement in two stages (a 37 °C incubation for 10 minutes for the BAP and an additional 4 °C incubation for seven days for the d-ROMs) (Fig. 2d). A good agreement between the conventional and the current method was confirmed for the hemolyzed serum (Table S3).

Using those conditions, the recovery rate in diluted samples was evaluated. As shown in Table S4, the recovery rates ranged from 85% to 113% for the d-ROMs assay and from 89% to 110% for the BAP assay, indicating acceptable analytical accuracy.

### 3.2 Positional effect across 384-well plate

As shown in Fig. S3a, we observed a horizontal gradient where absorbance values were higher in the left-side columns for both d-ROMs and BAP. Each of the three assays consisted of five or six columns, and the effect of column position within an assay ranged from 0.6% to 1.3% for d-ROMs and from 0.8% to 1.5% for BAP when median values for each column were compared (16 wells per column). In contrast, no such positional trend was observed across the rows (Fig. S3b).

As shown in Fig. S3c, the heat maps confirmed no apparent edge effects across the plate. Although there were some random outliers, the intra-assay coefficient of variation (CV) remained at 2–4% for d-ROMs and 3% for BAP, indicating a minimal impact of edge effects and evaporation.

### 3.3 Quality control strategy

**Fig. 3:**
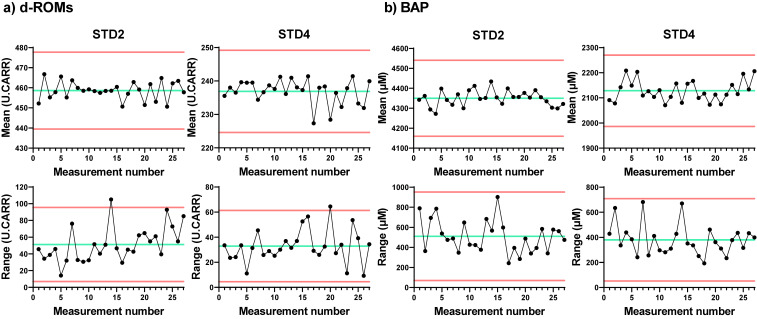
$\bar{X}$ and R control charts for d-ROMs and BAP measurements. Two levels of standard sera, STD2 (460 U.CARR, 4,328 µM) and STD4 (230 U.CARR, 2,164 µM), were monitored to evaluate the analytical stability of d-ROMs (a) and BAP (b) via concentration calculation using calibration curves from adjacent assays (eight readings/day for each STD2 and STD4). STD2 and STD4 in columns 1, 4, 5, 13, 16, and 17 were calculated using adjacent assays on the right (assay 2 or 3), and the rest referred to the one on the left (assay 1 or 2). The $\bar{X}$ charts display the daily mean values, and the R charts represent the difference between the maximum and minimum values within each day (the upper and lower rows, respectively, for 27 nonconsecutive days). Center lines (green) correspond to the overall mean ( $\bar{\bar{X}}$ ) and the average range ( $\bar{R}$ ). The upper and lower control limits (UCL and LCL), indicated by red lines, were calculated using standard $\bar{\bar{X}}$ -R chart constants for n = 8 (A_2_ = 0.373, D_3_ = 0.136, and D_4_ = 1.864) with the following formulas: $\bar{X}$ chart UCL and LCL = $\bar{\bar{X}}$ ± (A_2_ × $\bar{R}$ ), UCL for R chart = D_4_ × $\bar{R}$ , LCL for R chart = D_3_ × $\bar{R}$ .

As illustrated in Fig. 3a, the $\bar{X}$ values for both STD2 and STD4 remained consistently within the control limits across all 27 measurements for d-ROMs; however, the R values for each standard exceeded the control limit on one occasion. In contrast, the BAP assay showed good stability, with all $\bar{X}$ and R values for both standards remaining within the control limits throughout the entire evaluation period (Fig. 3b).

### 3.4 Inter-method agreement

**Fig. 4:**
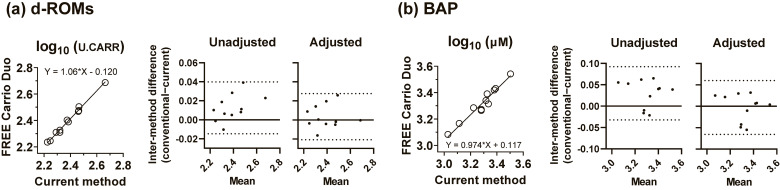
Inter-method agreement. d-ROMs (a) and BAP (b) levels were obtained for 11 serum samples without obvious turbidity using both the conventional method (FREE Carrio Duo) and the current small-scale method. Using log-transformed values, Deming regression yielded a slope of 1.06 (95% confidence interval, CI: 0.99–1.13) and an intercept of −0.12 (95% CI: −0.28 to 0.04) for log_10_(d-ROMs) and a slope of 0.97 (95% CI: 0.821–1.13) and an intercept of 0.117 (95% CI: −0.391 to 0.625) for log_10_(BAP). In the Bland–Altman plots, the difference between the two methods’ measurements was plotted against their mean for each sample (n = 11). The mean difference (i.e., the bias) and standard deviation (SD) were 0.013 and 0.014 for log_10_(d-ROMs) and 0.030 and 0.032 for log_10_(BAP) in the “Unadjusted” panel, respectively. The dashed lines indicate the 95% limits of agreement (mean difference ± 1.96 SD), which are −0.015 to 0.040 for log_10_(d-ROMs) and −0.032 to 0.092 for log_10_(BAP). The “Adjusted” plots used data corrected using the Deming regression equations (y = 1.06x − 0.120 for d-ROMs and y = 0.974x + 0.117 for BAP) to align with the FREE Carrio Duo values. The mean difference and SD were 0.0034 and 0.012 for log_10_(d-ROMs), −0.0027 and 0.032 for log_10_(BAP) in the “Adjusted” panel, respectively. The 95% limits of agreement were −0.021 to 0.028 for log_10_(d-ROMs) and −0.066 to 0.060 for log_10_(BAP).

As shown in Fig. 4, Deming regression analysis demonstrated good linearity between the conventional method (FREE Carrio Duo) and the current method for both markers; as described in the figure legend, the 95% confidence intervals for the slope crossed 1, and the intercepts crossed 0, suggesting the absence of proportional bias. However, visual inspection of the Bland–Altman plots in the “Unadjusted” panel for d-ROMs shows an increasing trend in inter-method differences with the mean. To adjust for this trend, we corrected the current method data to the conventional method data using Deming regression equations. As shown in the “Adjusted” panel, this correction eliminated the proportional trend and centered the differences evenly around zero, yielding a mean bias of 0.0034 (95% limits of agreement, LoA: −0.021 to 0.028) for log_10_(d-ROMs) and −0.0027 (95% LoA: −0.066 to 0.060) for log_10_(BAP). Thus, the biases and 95% LoA for d-ROMs and BAP support the clinical interchangeability of these two methods.

### 3.5 Reproducibility

Reproducibility is summarized in Table S5. CV for the intra-assay, inter-assay, and inter-day reproducibility ranged from 1% to 9% and 5% to 9%, and 6% to 8% for d-ROMs test. For BAP test, CV for the intra-assay, inter-assay, and inter-day ranged from 2% to 17%, 3% to 11%, and 3% to 10%. Inter-operator variations ranged from 4% to 7% for d-ROMs and 8% to 9% for BAP (Table S6).

### 3.6 Stability effect of sample storage conditions

**Fig. 5:**
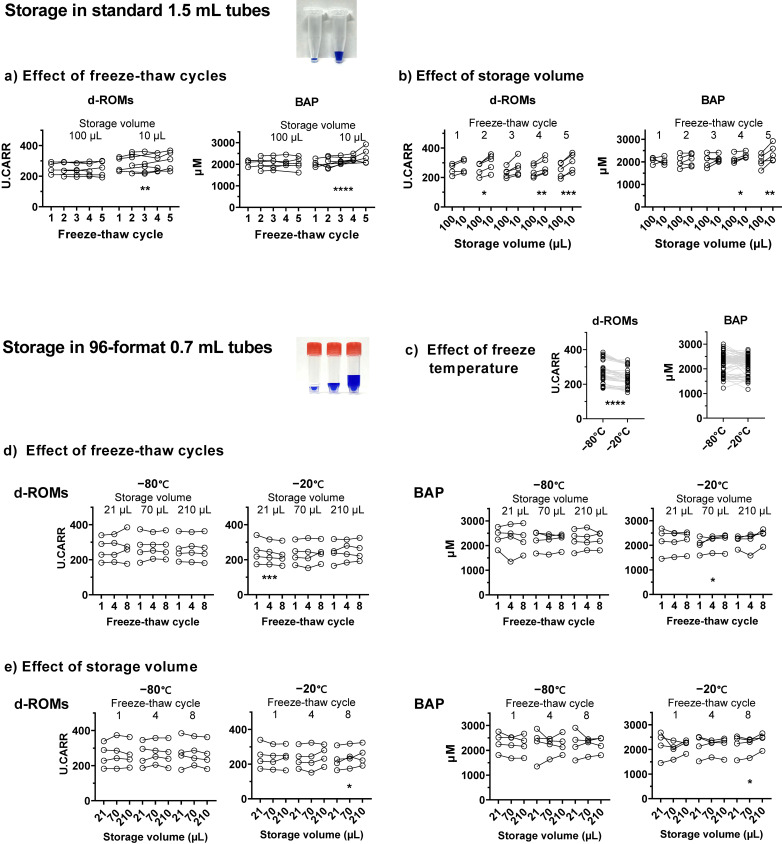
Effect of serum storage conditions on d-ROMs and BAP value. Serum samples were stored in either standard 1.5 mL tubes (seven normal sera from seven individuals) or 96-format 0.7 mL tubes (four normal sera from four individuals) to evaluate the impact of storage conditions. a) Effect of freeze-thaw cycles (one to five cycles at −80 °C and 25 °C) across different storage volumes. b) Comparison between different volumes (10 and 100 µL) within each freeze-thaw cycle. c) Effect of freezer temperature (−80 °C or −20 °C) within the same conditions from nine different conditions: three different freeze-thaw cycle numbers (one, four, and eight) × three different storage volumes (21, 70, and 210 µL). d) Effect of freeze-thaw cycles (one to eight cycles at −80 °C or −20 °C and 25 °C) across different storage volumes. e) Comparison between different volumes (21, 70, and 210 µL) within each freeze-thaw cycle. Data points represent the mean of triplicate (a, b) or duplicate (c, d) measurements. *p < 0.05, **p < 0.01, ***p < 0.001, ****p < 0.0001, using mixed-effects models (see Table S7 and SAS code in supplementary materials).

In standard 1.5 mL tubes, d-ROMs and BAP values remained stable across one to five freeze-thaw cycles when stored at a volume of 100 µL. In contrast, a positive trend was observed at a volume of 10 µL (Fig. 5a), with a significant interaction effect identified for BAP values (*P* for interaction = 0.016, Table S7). Both markers tended to yield higher values at 10 µL compared to 100 µL (Fig. 5b). When using 96-format 0.7 mL tubes, d-ROMs values were lower in samples stored at −20 °C than in those at −80 °C (Fig. 5c and Table S7), showing a downward trend compared to values obtained via the conventional method (*p* = 0.06, Student’s *t*-test). Notably, neither the number of freeze-thaw cycles nor the storage volume significantly impacted d-ROMs or BAP levels when samples were maintained at −80 °C (Figs. 5d and 5e).

## 4. Discussion

We have developed a high-throughput, cost-efficient in-house system for measuring d-ROMs and BAP, designed to support research applications. The system enables the simultaneous analysis of 144 serum samples in a single run by integrating a pipetting robot with a 384-well microplate format. However, a horizontal gradient of higher absorbance in the left column, where the samples are dispensed first, is unavoidable. We minimized the effect of this gradient by dividing the plate into three sections for d-ROMs and BAP analysis (six sections total). With several more optimizations, the assay exhibited good linearity, reproducibility, recovery, and agreement with the conventional FREE Carrio Duo analyzer. These results demonstrate that our system provides analytical precision comparable to the conventional method.

The integration of $\bar{X}$ -R control charts in the current protocol provide a robust framework for ensuring long-term analytical stability and consistent data quality. Under this framework, $\bar{X}$ chart outliers should be interpreted as potential indicators of systemic bias, such as errors in data entry or machine trouble. Conversely, R-chart outliers are more likely to suggest increased random error, potentially reflecting mechanical issues such as robotic pipetting drift, the presence of air bubbles, or evaporation due to sealing failures. For a practical guide, we propose a quality control checklist and an operational flowchart, as shown in Fig. S4. First, the linearity of the independent standard curves must be verified, with a coefficient of determination (R^2^) ≥0.99 as the minimum requirement. Second, the $\bar{X}$ -R control charts should show the values within the predefined control limits. Third, each unknown sample must be checked to confirm that its measured value falls within the linear range of the standard curve (≤575 U.CARR or ≤5,410 µM). If any of these criteria are not met, the assay should be flagged as a failure.

The required sample volume was reduced by 83%, while the required volumes of d-ROMs and BAP reagents were reduced by 93% and 86%, respectively. Consequently, the current method conserves precious biological samples and reduces overall testing costs. Compared to the total cost per 1,000 samples for the conventional method, which includes a labor requirement of 167 hours, the total cost for the current method, which includes 35 hours of labor, was reduced by approximate 86%. Although the final color measurement for d-ROMs is taken after one week, a 79% reduction in labor time was achieved.

In clinical settings, serum samples often appear cloudy due to elevated triglyceride levels [19]. Such lipemic samples are known to interfere with colorimetric assays [20, 21], which can affect the accuracy of test results. For this reason, the conventional system recommends avoiding postprandial sample collection to prevent underestimation of BAP values. While this could limit the application of the system, our method offers a solution, as the system is more robust to this problem due to the additional extraction step.

In large-scale studies, sample preservation is a critical issue due to concerns about sample degradation. Our findings suggest that 100 µL of serum is less susceptible to degradation during cryopreservation than 10 µL of serum when using standard 1.5 mL tubes. This may be because lipid oxidation and endogenous antioxidant degradation caused by freeze-thaw cycles [22] are accelerated in smaller volumes of stored serum, possibly due to increased exposed surface area and reduced buffering capacity [23]. 0.7 mL tubes allow for the storage of as little as 21 µL of serum—a volume representing only 3% of the total tube capacity. When stored at −80 °C in these tubes, d-ROMs and BAP levels could be kept without being affected by up to eight freeze-thaw cycles. One reasonable explanation would be the structural advantages of the 0.7 mL tube: its elongated shape minimizes the surface area exposed to air, and the screw-cap design ensures an airtight seal against the external environment.

While the present method offers improved applicability for large-scale studies, several limitations should be acknowledged. One limitation of the current method is that the final colorimetric measurement for d-ROMs analysis is taken a week later, which restricts its use in applications requiring rapid processing. However, it is challenging to analyze exclusively clear, non-lipemic serum samples in epidemiological studies, because a certain percentage of individuals have high triglyceride concentrations even in a fasting state [24]. Therefore, to broaden the method’s applicability, measurement after a one-week incubation period is recommended. Future research aims to optimize and, if possible, shorten the incubation time to enhance applicability. Additionally, the mechanism by which an additional centrifugation step improves linearity remains unclear. This uncertainty may hinder further methodological improvements. The limited number of lipemic and hemolytic samples, as well as the small sample size (n = 11) for the inter-method agreement analysis, also constitute limitations of this study. Although the absence of proportional bias between the two methods was statistically suggested, it may have been undetectable due to the small sample size. Therefore, we recommend using a correction value based on the Deming regression equation (see Fig. 4 and the conclusion section).

## 5. Conclusion

We have developed a high-throughput, cost-efficient in-house system for the simultaneous measurement of d-ROMs and BAP using a pipetting robot and 384-well plates. The system was optimized to include lipemic serum samples by incorporating two methodological modifications. First, a supernatant extraction step was added for BAP. Second, a two-step color measurement was implemented, in which BAP was measured after a 10-minute incubation at 37 °C, followed by d-ROMs measurement after a seven-day incubation at 4 °C. Both d-ROMs and BAP assays demonstrated strong linearity and reproducibility, with minimal variation across repeated measurements. The system demonstrated strong agreement with the conventional FREE Carrio Duo analyzer, as demonstrated by the following regression equations:

$$\begin{array}{rl} & {Log}_{10}(\text{d-ROMs},\text{conventional method})\\ & \;=1.06\times {Log}_{10}(\text{d-ROMs},\text{current method})-0.12 \end{array}$$

$$\begin{array}{rl} & {Log}_{10}(\text{BAP},\text{conventional method})\\ & \;=0.974\times {Log}_{10}(\text{BAP},\text{current method})+0.117 \end{array}$$

The system has significantly reduced both sample and reagent volumes, as well as measurement time. These improvements make the method both efficient and cost-effective, supporting precise and large-scale analysis of oxidative stress.

## Abbreviations

ROS: Reactive oxygen species
d-ROMs: Diacron-reactive oxygen metabolites
BAP: Biological antioxidant potential
OD: Optical density
STD: Standard serum
U.CARR: Carratelli Units
CV: Coefficient of variation

## Supplementary information

**Additional file 1:** SAS scripts for each statistical analysis.

**Additional file 2:** Andrew pipetting protocol_d-ROMs.

**Additional file 3:** Andrew pipetting protocol_BAP-1.

**Additional file 4:** Andrew pipetting protocol_BAP-2.

**Additional file 5:** Plate layout templates.

**Additional file 6:** **Figure S1:** Procedure for small-scale d-ROMs and BAP measurement. **Figure S2.** Freezing and thawing of serum for sample stability test. **Table S1.** Reproducibility of lipemic serum. **Table S2.** Calibration stability. **Table S3.** Reproducibility of hemolyzed serum. **Table S4.** Recovery rate. **Figure S3.** Intra-plate variations in 384-well microplate assays. **Table S5.** Reproducibility of normal serum. **Table S6.** Inter-operator variation assay. **Table S7.** Effects of storage conditions. **Figure S4.** Quality control framework and decision-making flowchart for d-ROMs and BAP assays.

## Declarations

### Availability of data and materials

All human serum samples used in this study were irreversibly anonymized prior to experimental analysis by assigning random identification numbers. All data were handled in accordance with institutional guidelines and stored on secure, access-restricted servers. The analyzed data were used exclusively for research purposes and were not accessible to unauthorized individuals. Data sharing is limited to anonymized, aggregated datasets and will be made available upon reasonable request, subject to approval by the relevant ethics committee and in compliance with institutional and national regulations.

## References

[1] Sies H, Berndt C, Jones DP. Oxidative stress. Annu Rev Biochem. 2017;86:715–48. 28441057 10.1146/annurev-biochem-061516-045037

[2] Scandalios JG. Oxidative stress responses—what have genome-scale studies taught us? Genome Biol. 2002;3:1019. 10.1186/gb-2002-3-7-reviews1019 PMC139384 12184812

[3] Birben E, Sahiner UM, Sackesen C, Erzurum S, Kalayci O. Oxidative stress and antioxidant defense. World Allergy Organ J. 2012;5:9–19. 23268465 10.1097/WOX.0b013e3182439613 PMC3488923

[4] Sharifi-Rad M, Anil Kumar NV, Zucca P, Varoni EM, Dini L, Panzarini E, Rajkovic J, Tsouh Fokou PV, Azzini E, Peluso I, Prakash Mishra A, Nigam M, El Rayess Y, Beyrouthy ME, Polito L, Iriti M, Martins N, Martorell M, Docea AO, Setzer WN, Calina D, Cho WC, Sharifi-Rad J. Lifestyle, oxidative stress, and antioxidants: back and forth in the pathophysiology of chronic diseases. Front Physiol. 2020;11:694. 32714204 10.3389/fphys.2020.00694 PMC7347016

[5] Husain S, Hillmann K, Hengst K, Englert H. Effects of a lifestyle intervention on the biomarkers of oxidative stress in non-communicable diseases: A systematic review. Front Aging. 2023;4:1085511. 36970730 10.3389/fragi.2023.1085511 PMC10034086

[6] Vatner SF, Zhang J, Oydanich M, Berkman T, Naftalovich R, Vatner DE. Healthful aging mediated by inhibition of oxidative stress. Ageing Res Rev. 2020;64:101194. 33091597 10.1016/j.arr.2020.101194 PMC7710569

[7] Wu LL, Chiou CC, Chang PY, Wu JT. Urinary 8-OHdG: A marker of oxidative stress to DNA and a risk factor for cancer, atherosclerosis and diabetics. Clin Chim Acta. 2004;339:1–9. 14687888 10.1016/j.cccn.2003.09.010

[8] Janero DR. Malondialdehyde and thiobarbituric acid-reactivity as diagnostic indices of lipid peroxidation and peroxidative tissue injury. Free Radic Biol Med. 1990;9:515–40. 2079232 10.1016/0891-5849(90)90131-2

[9] Crespo-Sanjuán J, Calvo-Nieves MD, Aguirre-Gervás B, Herreros-Rodríguez J, Velayos-Jiménez B, Castro-Alija MJ, Muñoz-Moreno MF, Sánchez D, Zamora-González N, Bajo-Grañeras R, García-Centeno RM, Largo Cabrerizo ME, Bustamante MR, Garrote-Adrados JA. Early detection of high oxidative activity in patients with adenomatous intestinal polyps and colorectal adenocarcinoma: myeloperoxidase and oxidized low-density lipoprotein in serum as new markers of oxidative stress in colorectal cancer. Lab Med. 2015;46:123–35. 25918191 10.1309/LMZJJU6BC86WUDHW

[10] Saribal D, Hocaoglu-Emre FS, Karaman F, Mırsal H, Akyolcu MC. Trace element levels and oxidant/antioxidant status in patients with alcohol abuse. Biol Trace Elem Res. 2020;193:7–13. 30805875 10.1007/s12011-019-01681-y

[11] Bacchetti T, Simonetti O, Ricotti F, Offidani A, Ferretti G. Plasma oxidation status and antioxidant capacity in psoriatic children. Arch Dermatol Res. 2020;312:33–9. 31531730 10.1007/s00403-019-01976-z

[12] Kitaoka T, Morimoto M, Hashimoto T, Tsuda Y, Nakatsu T, Kyotani S. Evaluation of the efficacy of drug treatment based on measurement of the oxidative stress, using reactive oxygen metabolites and biological antioxidant potential, in children with autism spectrum disorder and attention deficit hyperactivity disorder. J Pharm Health Care Sci. 2020;6:8. 32351702 10.1186/s40780-020-00164-w PMC7183642

[13] Morimoto M, Hashimoto T, Tsuda Y, Kitaoka T, Kyotani S. Evaluation of oxidative stress and antioxidant capacity in healthy children. J Chin Med Assoc. 2019;82:651–4. 30893262 10.1097/JCMA.0000000000000045 PMC13047978

[14] Kilk K, Meitern R, Härmson O, Soomets U, Hõrak P. Assessment of oxidative stress in serum by d-ROMs test. Free Radic Res. 2014;48:883–9. 24773038 10.3109/10715762.2014.919390

[15] Dohi K, Satoh K, Ohtaki H, Shioda S, Miyake Y, Shindo M, Aruga T. Elevated plasma levels of bilirubin in patients with neurotrauma reflect its pathophysiological role in free radical scavenging. In Vivo. 2005;19:855–60. 16097438

[16] Ichikawa G, Negishi Y, Tsuchiya R, Higuchi L, Shiraishi T, Ikeda M, Kaseki H, Morita R, Suzuki S. Oxidative stress and antioxidant capacity in patients with endometrioma. J Nippon Med Sch. 2024;91:146–54. 38432930 10.1272/jnms.JNMS.2024_91-204

[17] Yagi H, Sumino H, Yoshida K, Aoki T, Tsunekawa K, Araki O, Kimura T, Nara M, Nakajima K, Murakami M. Biological antioxidant potential negatively correlates with carotid artery intima-media thickness. Int Heart J. 2016;57:220–5. 26973274 10.1536/ihj.15-389

[18] Fukui T, Yamauchi K, Maruyama M, Yasuda T, Kohno M, Abe Y. Significance of measuring oxidative stress in lifestyle-related diseases from the viewpoint of correlation between d-ROMs and BAP in Japanese subjects. Hypertens Res. 2011;34:1041–5. 21677660 10.1038/hr.2011.76

[19] Raturi M, Kusum A. Deciphering the reasons for milky-white blood donor plasma. Transfus Clin Biol. 2020;27:259–61. 32890730 10.1016/j.tracli.2020.08.006

[20] Kroll MH. Evaluating interference caused by lipemia. Clin Chem. 2004;50:1968–9. 15502078 10.1373/clinchem.2004.038075

[21] Ji JZ, Meng QH. Evaluation of the interference of hemoglobin, bilirubin, and lipids on Roche Cobas 6000 assays. Clin Chim Acta. 2011;412:1550–3. 21575617 10.1016/j.cca.2011.04.034

[22] Paltiel L, Rønningen KS, Meltzer HM, Baker SV, Hoppin JA. Evaluation of freeze-thaw cycles on stored plasma in the biobank of the Norwegian mother and child cohort study. Cell Preserv Technol. 2008;6:223–9. 20428472 10.1089/cpt.2008.0012 PMC2860294

[23] Gislefoss RE, Lauritzen M, Langseth H, Mørkrid L. Effect of multiple freeze-thaw cycles on selected biochemical serum components. Clin Chem Lab Med. 2017;55:967–73. 27987362 10.1515/cclm-2016-0892

[24] Paragh G, Németh Á, Harangi M, Banach M, Fülöp P. Causes, clinical findings and therapeutic options in chylomicronemia syndrome, a special form of hypertriglyceridemia. Lipids Health Dis. 2022;21:21. 35144640 10.1186/s12944-022-01631-z PMC8832680

