# Supplementary material for: High-throughput, low volume d-ROMs and BAP assays: 384-well plate method for large-scale studies
Source: Environ Health Prev Med. 2026 Jul 3;31:42. doi: 10.1265/ehpm.25-00354 (PMC13366183; doi:10.1265/ehpm.25-00354)
Supplement: Supplementary file 1 — Additional file 1: SAS scripts for each statistical analysis. [file ehpm-31-042-s001.docx]

Supplementary file 1: SAS scripts for each statistical analysis

**Storage in standard 1.5 mL tubes**

**Variable name:**

*“volume”*: storage volume, *“freezethaw”*: number of freeze-thaw cycles, *“id”*: serum identification number

**For Table S5**

- ***proc*** ***mixed****; class id; model ln(d-ROMs) = volume freezethaw volume*freezethaw/s; random = id;* ***run****;*
- ***proc*** ***mixed****; class id; model ln(BAP) = volume freezethaw volume*freezethaw/s; random = id ;* ***run****;*

**For Figure 5a**

- ***proc sort****; by volume;* ***proc*** ***mixed****; by volume; class id; model ln(d-ROMs) = freezethaw/s; random = id;* ***run****;*
- ***proc sort****; by volume;* ***proc*** ***mixed****; by volume; class id; model ln(BAP) = freezethaw/s; random = id;* ***run****;*

**For Figure 5b**

- ***proc sort****; by freezethaw;* ***proc*** ***mixed****; by freezethaw; class id; model ln(d-ROMs) = volume/s; random = id;* ***run****;*
- ***proc sort****; by freezethaw;* ***proc*** ***mixed****; by freezethaw; class id; model ln(BAP) = volume/s; random = id;* ***run****;*

**Storage in 96-format 0.7 mL tubes**

**Variable name:**

*“volume3”*: storage volume in three ranks, *“freezethaw3”*: number of freeze-thaw cycles in three ranks, *“id”*: serum identification number, *“temp”*: freeze temperature

**For Table S5**

- ***proc*** ***mixed****; class id; model ln(d-ROMs) = volume3 freezethaw3 volume3*freezethaw3 temp/s; random = id;* ***run****;*
- ***proc sort****; by temp;* ***proc*** ***mixed****; by temp; class id; model ln(d-ROMs) = volume3 freezethaw3 volume3*freezethaw3/s; random = id;* ***run****;*
- ***proc*** ***mixed****; class id; model ln(BAP) = volume3 freezethaw3 volume3*freezethaw3 temp/s; random = id;* ***run****;*
- ***proc sort****; by temp;* ***proc*** ***mixed****; by temp; class id; model ln(BAP) = volume3 freezethaw3 volume3*freezethaw3/s; random = id;* ***run****;*

**For Figure 5c**

- ***proc*** ***mixed****; class id; model ln(d-ROMs) = volume3 freezethaw3 volume3*freezethaw3 temp/s; random = id;* ***run****;*
- ***proc*** ***mixed****; class id; model ln(BAP) = volume3 freezethaw3 volume3*freezethaw3 temp/s; random = id;* ***run****;*

**For Figure 5d**

- ***proc sort****; by temp volume3;* ***proc*** ***mixed****; by temp volume3; class id; model ln(d-ROMs) = freezethaw3/s; random = id;* ***run****;*
- ***proc sort****; by temp volume3;* ***proc*** ***mixed****; by temp volume3; class id; model ln(BAP) = freezethaw3/s; random = id;* ***run****;*

**For Figure 5e**

- ***proc sort****; by temp freezethaw3;* ***proc*** ***mixed****; by temp freezethaw3; class id; model ln(d-ROMs) = volume3/s; random = id;* ***run****;*
- ***proc sort****; by temp freezethaw3;* ***proc*** ***mixed****; by temp freezethaw3; class id; model ln(BAP) = volume3/s; random = id;* ***run****;*
