# Supplementary figures and images for: High-throughput, low volume d-ROMs and BAP assays: 384-well plate method for large-scale studies
Source: Environ Health Prev Med. 2026 Jul 3;31:42. doi: 10.1265/ehpm.25-00354 (PMC13366183; doi:10.1265/ehpm.25-00354)

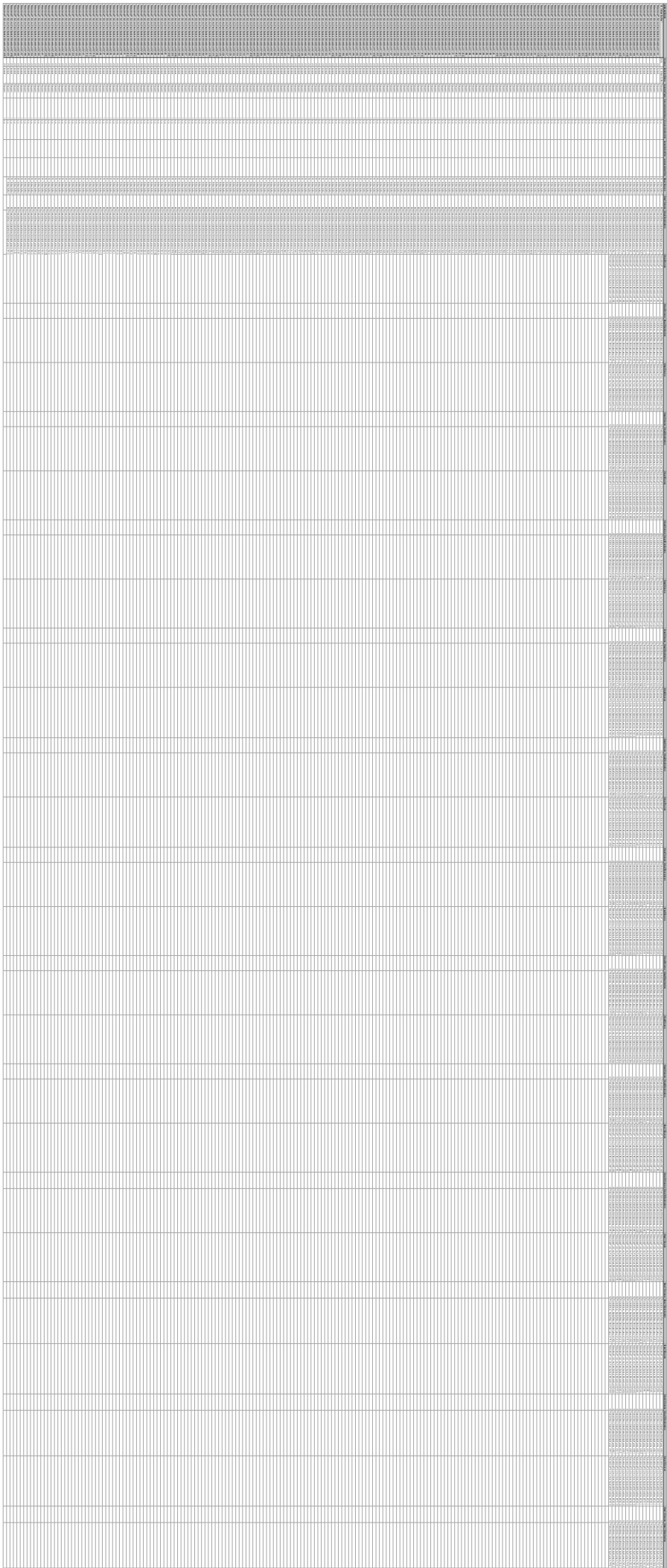

Supplement: Supplementary file 2 — Additional file 2: Andrew pipetting protocol_d-ROMs. [file ehpm-31-042-s002.pdf]

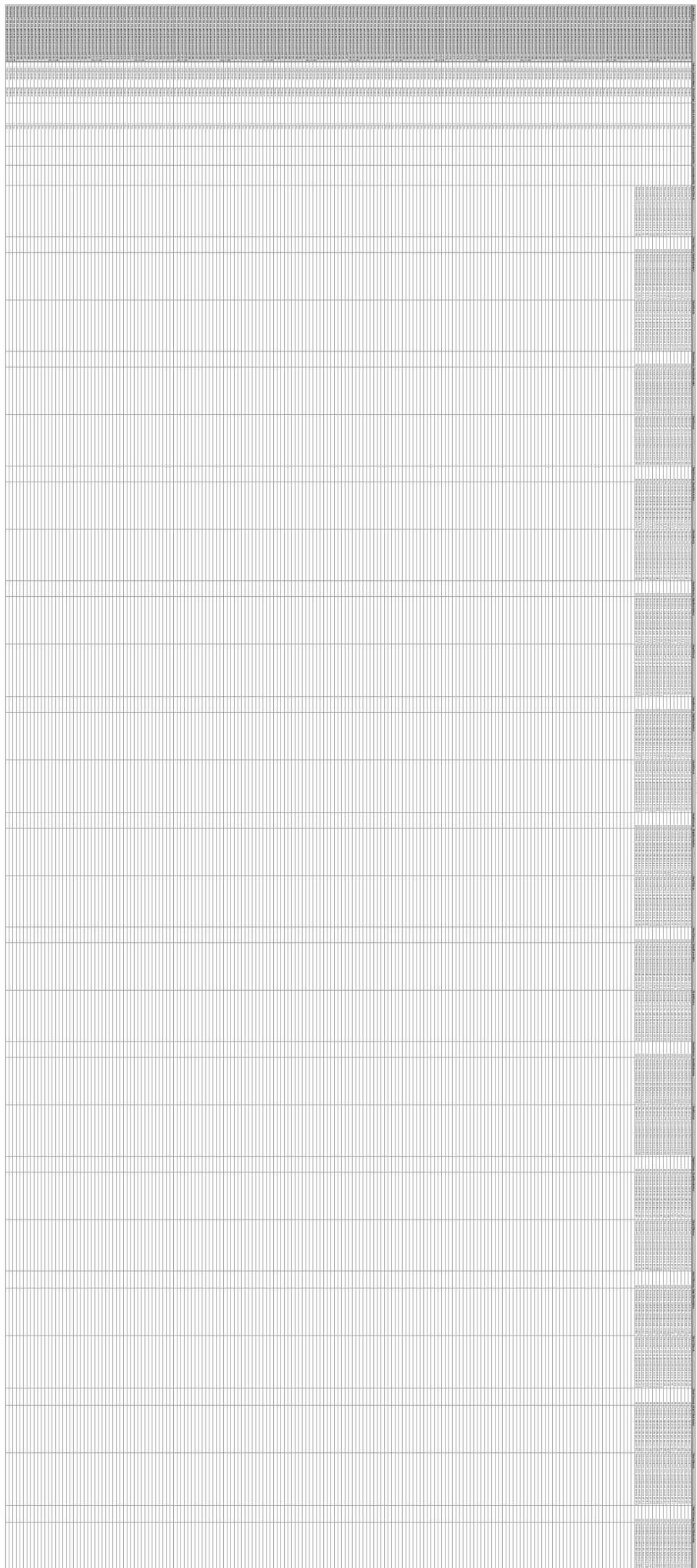

Supplement: Supplementary file 4 — Additional file 4: Andrew pipetting protocol_BAP-2. [file ehpm-31-042-s004.pdf]
