# Supplementary material for: High-throughput, low volume d-ROMs and BAP assays: 384-well plate method for large-scale studies
Source: Environ Health Prev Med. 2026 Jul 3;31:42. doi: 10.1265/ehpm.25-00354 (PMC13366183; doi:10.1265/ehpm.25-00354)
Supplement: Supplementary file 5 — Additional file 5: Plate layout templates. [file ehpm-31-042-s005.pptx]

## Slide 1
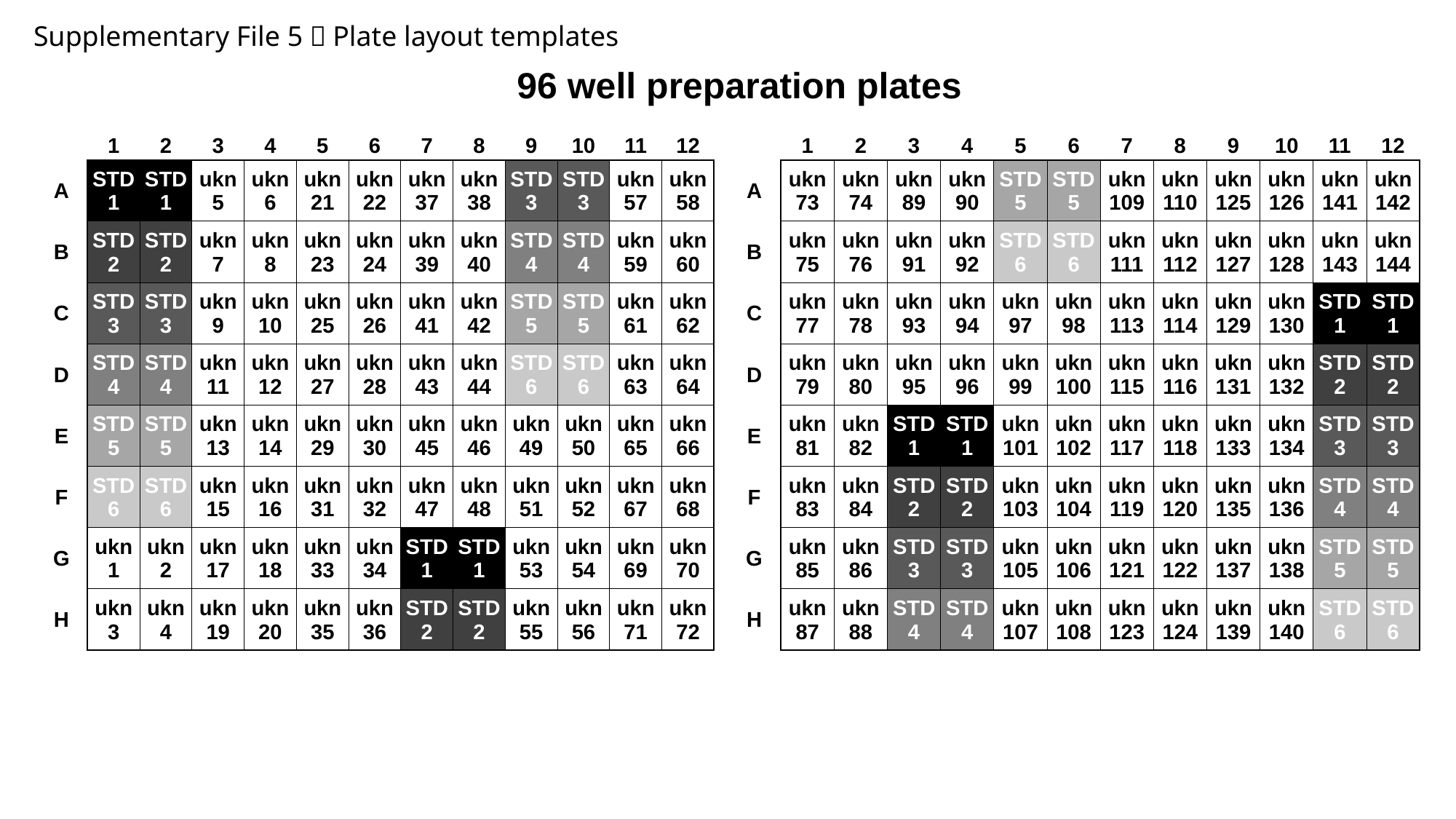

Supplementary File 5：Plate layout templates
96 well preparation plates
| | 1 | 2 | 3 | 4 | 5 | 6 | 7 | 8 | 9 | 10 | 11 | 12 |
| --- | --- | --- | --- | --- | --- | --- | --- | --- | --- | --- | --- | --- |
| A | STD 1 | STD 1 | ukn 5 | ukn 6 | ukn 21 | ukn 22 | ukn 37 | ukn 38 | STD 3 | STD 3 | ukn 57 | ukn 58 |
| B | STD 2 | STD 2 | ukn 7 | ukn 8 | ukn 23 | ukn 24 | ukn 39 | ukn 40 | STD 4 | STD 4 | ukn 59 | ukn 60 |
| C | STD 3 | STD 3 | ukn 9 | ukn 10 | ukn 25 | ukn 26 | ukn 41 | ukn 42 | STD 5 | STD 5 | ukn 61 | ukn 62 |
| D | STD 4 | STD 4 | ukn 11 | ukn 12 | ukn 27 | ukn 28 | ukn 43 | ukn 44 | STD 6 | STD 6 | ukn 63 | ukn 64 |
| E | STD 5 | STD 5 | ukn 13 | ukn 14 | ukn 29 | ukn 30 | ukn 45 | ukn 46 | ukn 49 | ukn 50 | ukn 65 | ukn 66 |
| F | STD 6 | STD 6 | ukn 15 | ukn 16 | ukn 31 | ukn 32 | ukn 47 | ukn 48 | ukn 51 | ukn 52 | ukn 67 | ukn 68 |
| G | ukn 1 | ukn 2 | ukn 17 | ukn 18 | ukn 33 | ukn 34 | STD 1 | STD 1 | ukn 53 | ukn 54 | ukn 69 | ukn 70 |
| H | ukn 3 | ukn 4 | ukn 19 | ukn 20 | ukn 35 | ukn 36 | STD 2 | STD 2 | ukn 55 | ukn 56 | ukn 71 | ukn 72 |
| | 1 | 2 | 3 | 4 | 5 | 6 | 7 | 8 | 9 | 10 | 11 | 12 |
| --- | --- | --- | --- | --- | --- | --- | --- | --- | --- | --- | --- | --- |
| A | ukn 73 | ukn 74 | ukn 89 | ukn 90 | STD 5 | STD 5 | ukn 109 | ukn 110 | ukn 125 | ukn 126 | ukn 141 | ukn 142 |
| B | ukn 75 | ukn 76 | ukn 91 | ukn 92 | STD 6 | STD 6 | ukn 111 | ukn 112 | ukn 127 | ukn 128 | ukn 143 | ukn 144 |
| C | ukn 77 | ukn 78 | ukn 93 | ukn 94 | ukn 97 | ukn 98 | ukn 113 | ukn 114 | ukn 129 | ukn 130 | STD 1 | STD 1 |
| D | ukn 79 | ukn 80 | ukn 95 | ukn 96 | ukn 99 | ukn 100 | ukn 115 | ukn 116 | ukn 131 | ukn 132 | STD 2 | STD 2 |
| E | ukn 81 | ukn 82 | STD 1 | STD 1 | ukn 101 | ukn 102 | ukn 117 | ukn 118 | ukn 133 | ukn 134 | STD 3 | STD 3 |
| F | ukn 83 | ukn 84 | STD 2 | STD 2 | ukn 103 | ukn 104 | ukn 119 | ukn 120 | ukn 135 | ukn 136 | STD 4 | STD 4 |
| G | ukn 85 | ukn 86 | STD 3 | STD 3 | ukn 105 | ukn 106 | ukn 121 | ukn 122 | ukn 137 | ukn 138 | STD 5 | STD 5 |
| H | ukn 87 | ukn 88 | STD 4 | STD 4 | ukn 107 | ukn 108 | ukn 123 | ukn 124 | ukn 139 | ukn 140 | STD 6 | STD 6 |

## Slide 2
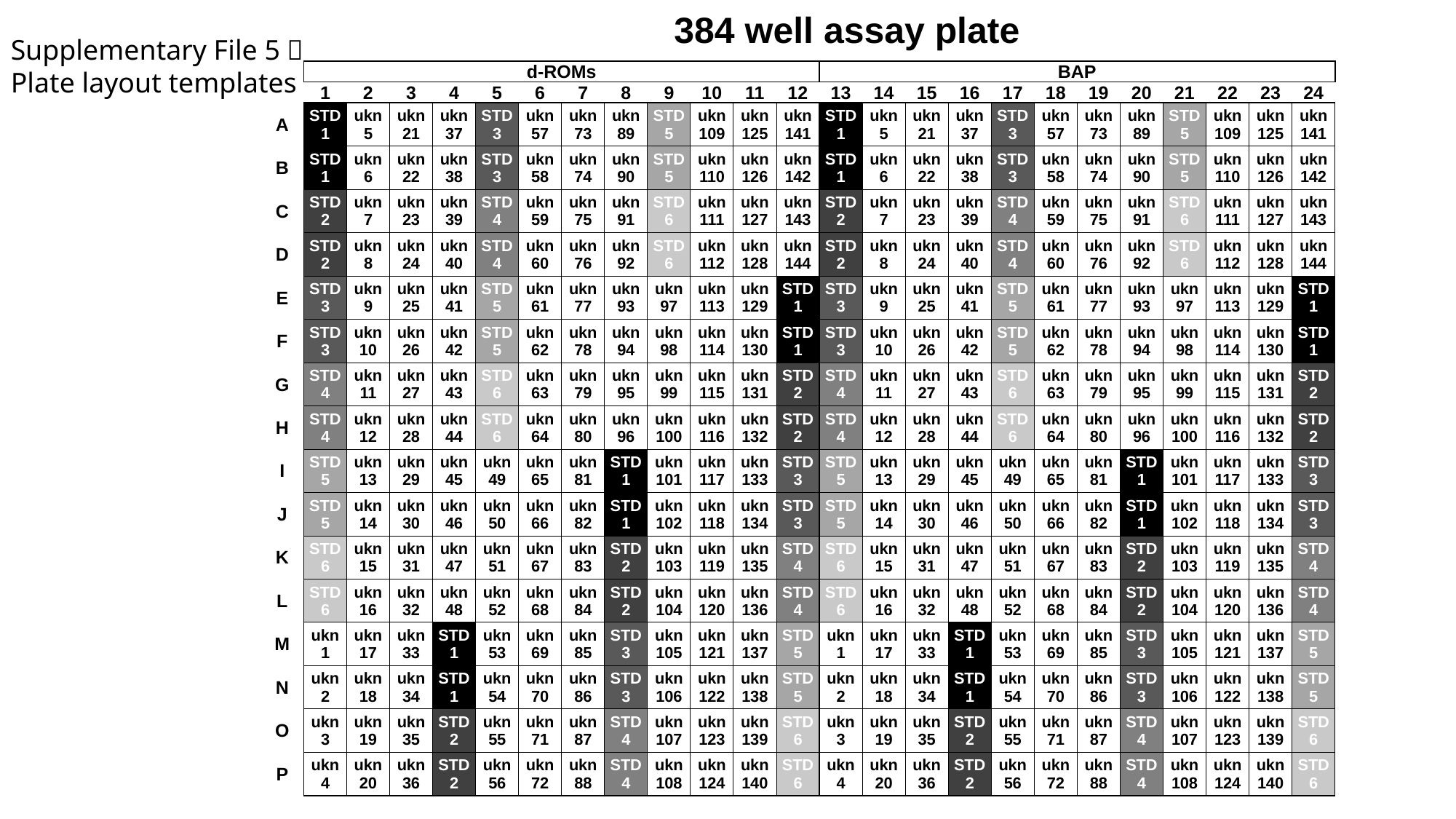

384 well assay plate
Supplementary File 5：
Plate layout templates
| | d-ROMs | | | | | | | | | | | | BAP | | | | | | | | | | | |
| --- | --- | --- | --- | --- | --- | --- | --- | --- | --- | --- | --- | --- | --- | --- | --- | --- | --- | --- | --- | --- | --- | --- | --- | --- |
| | 1 | 2 | 3 | 4 | 5 | 6 | 7 | 8 | 9 | 10 | 11 | 12 | 13 | 14 | 15 | 16 | 17 | 18 | 19 | 20 | 21 | 22 | 23 | 24 |
| A | STD 1 | ukn 5 | ukn 21 | ukn 37 | STD 3 | ukn 57 | ukn 73 | ukn 89 | STD 5 | ukn 109 | ukn 125 | ukn 141 | STD 1 | ukn 5 | ukn 21 | ukn 37 | STD 3 | ukn 57 | ukn 73 | ukn 89 | STD 5 | ukn 109 | ukn 125 | ukn 141 |
| B | STD 1 | ukn 6 | ukn 22 | ukn 38 | STD 3 | ukn 58 | ukn 74 | ukn 90 | STD 5 | ukn 110 | ukn 126 | ukn 142 | STD 1 | ukn 6 | ukn 22 | ukn 38 | STD 3 | ukn 58 | ukn 74 | ukn 90 | STD 5 | ukn 110 | ukn 126 | ukn 142 |
| C | STD 2 | ukn 7 | ukn 23 | ukn 39 | STD 4 | ukn 59 | ukn 75 | ukn 91 | STD 6 | ukn 111 | ukn 127 | ukn 143 | STD 2 | ukn 7 | ukn 23 | ukn 39 | STD 4 | ukn 59 | ukn 75 | ukn 91 | STD 6 | ukn 111 | ukn 127 | ukn 143 |
| D | STD 2 | ukn 8 | ukn 24 | ukn 40 | STD 4 | ukn 60 | ukn 76 | ukn 92 | STD 6 | ukn 112 | ukn 128 | ukn 144 | STD 2 | ukn 8 | ukn 24 | ukn 40 | STD 4 | ukn 60 | ukn 76 | ukn 92 | STD 6 | ukn 112 | ukn 128 | ukn 144 |
| E | STD 3 | ukn 9 | ukn 25 | ukn 41 | STD 5 | ukn 61 | ukn 77 | ukn 93 | ukn 97 | ukn 113 | ukn 129 | STD 1 | STD 3 | ukn 9 | ukn 25 | ukn 41 | STD 5 | ukn 61 | ukn 77 | ukn 93 | ukn 97 | ukn 113 | ukn 129 | STD 1 |
| F | STD 3 | ukn 10 | ukn 26 | ukn 42 | STD 5 | ukn 62 | ukn 78 | ukn 94 | ukn 98 | ukn 114 | ukn 130 | STD 1 | STD 3 | ukn 10 | ukn 26 | ukn 42 | STD 5 | ukn 62 | ukn 78 | ukn 94 | ukn 98 | ukn 114 | ukn 130 | STD 1 |
| G | STD 4 | ukn 11 | ukn 27 | ukn 43 | STD 6 | ukn 63 | ukn 79 | ukn 95 | ukn 99 | ukn 115 | ukn 131 | STD 2 | STD 4 | ukn 11 | ukn 27 | ukn 43 | STD 6 | ukn 63 | ukn 79 | ukn 95 | ukn 99 | ukn 115 | ukn 131 | STD 2 |
| H | STD 4 | ukn 12 | ukn 28 | ukn 44 | STD 6 | ukn 64 | ukn 80 | ukn 96 | ukn 100 | ukn 116 | ukn 132 | STD 2 | STD 4 | ukn 12 | ukn 28 | ukn 44 | STD 6 | ukn 64 | ukn 80 | ukn 96 | ukn 100 | ukn 116 | ukn 132 | STD 2 |
| I | STD 5 | ukn 13 | ukn 29 | ukn 45 | ukn 49 | ukn 65 | ukn 81 | STD 1 | ukn 101 | ukn 117 | ukn 133 | STD 3 | STD 5 | ukn 13 | ukn 29 | ukn 45 | ukn 49 | ukn 65 | ukn 81 | STD 1 | ukn 101 | ukn 117 | ukn 133 | STD 3 |
| J | STD 5 | ukn 14 | ukn 30 | ukn 46 | ukn 50 | ukn 66 | ukn 82 | STD 1 | ukn 102 | ukn 118 | ukn 134 | STD 3 | STD 5 | ukn 14 | ukn 30 | ukn 46 | ukn 50 | ukn 66 | ukn 82 | STD 1 | ukn 102 | ukn 118 | ukn 134 | STD 3 |
| K | STD 6 | ukn 15 | ukn 31 | ukn 47 | ukn 51 | ukn 67 | ukn 83 | STD 2 | ukn 103 | ukn 119 | ukn 135 | STD 4 | STD 6 | ukn 15 | ukn 31 | ukn 47 | ukn 51 | ukn 67 | ukn 83 | STD 2 | ukn 103 | ukn 119 | ukn 135 | STD 4 |
| L | STD 6 | ukn 16 | ukn 32 | ukn 48 | ukn 52 | ukn 68 | ukn 84 | STD 2 | ukn 104 | ukn 120 | ukn 136 | STD 4 | STD 6 | ukn 16 | ukn 32 | ukn 48 | ukn 52 | ukn 68 | ukn 84 | STD 2 | ukn 104 | ukn 120 | ukn 136 | STD 4 |
| M | ukn 1 | ukn 17 | ukn 33 | STD 1 | ukn 53 | ukn 69 | ukn 85 | STD 3 | ukn 105 | ukn 121 | ukn 137 | STD 5 | ukn 1 | ukn 17 | ukn 33 | STD 1 | ukn 53 | ukn 69 | ukn 85 | STD 3 | ukn 105 | ukn 121 | ukn 137 | STD 5 |
| N | ukn 2 | ukn 18 | ukn 34 | STD 1 | ukn 54 | ukn 70 | ukn 86 | STD 3 | ukn 106 | ukn 122 | ukn 138 | STD 5 | ukn 2 | ukn 18 | ukn 34 | STD 1 | ukn 54 | ukn 70 | ukn 86 | STD 3 | ukn 106 | ukn 122 | ukn 138 | STD 5 |
| O | ukn 3 | ukn 19 | ukn 35 | STD 2 | ukn 55 | ukn 71 | ukn 87 | STD 4 | ukn 107 | ukn 123 | ukn 139 | STD 6 | ukn 3 | ukn 19 | ukn 35 | STD 2 | ukn 55 | ukn 71 | ukn 87 | STD 4 | ukn 107 | ukn 123 | ukn 139 | STD 6 |
| P | ukn 4 | ukn 20 | ukn 36 | STD 2 | ukn 56 | ukn 72 | ukn 88 | STD 4 | ukn 108 | ukn 124 | ukn 140 | STD 6 | ukn 4 | ukn 20 | ukn 36 | STD 2 | ukn 56 | ukn 72 | ukn 88 | STD 4 | ukn 108 | ukn 124 | ukn 140 | STD 6 |
