# Supplementary material for: High-throughput, low volume d-ROMs and BAP assays: 384-well plate method for large-scale studies
Source: Environ Health Prev Med. 2026 Jul 3;31:42. doi: 10.1265/ehpm.25-00354 (PMC13366183; doi:10.1265/ehpm.25-00354)
Supplement: Supplementary file 6 — Additional file 6: Figure S1: Procedure for small-scale d-ROMs and BAP measurement. Figure S2. Freezing and thawing of serum for sample stability test. Table S1. Reproducibility of lipemic serum. Table S2. Calibration stability. Table S3. Reproducibility of hemolyzed serum. Table S4. Recovery rate. Figure S3. Intra-plate variations in 384-well microplate assays. Table S5. Reproducibility of normal serum. Table S6. Inter-operator variation assay. Table S7. Effects of storage conditions. Figure S4. Quality control framework and decision-making flowchart for d-ROMs and BAP assays. [file ehpm-31-042-s006.docx]

**
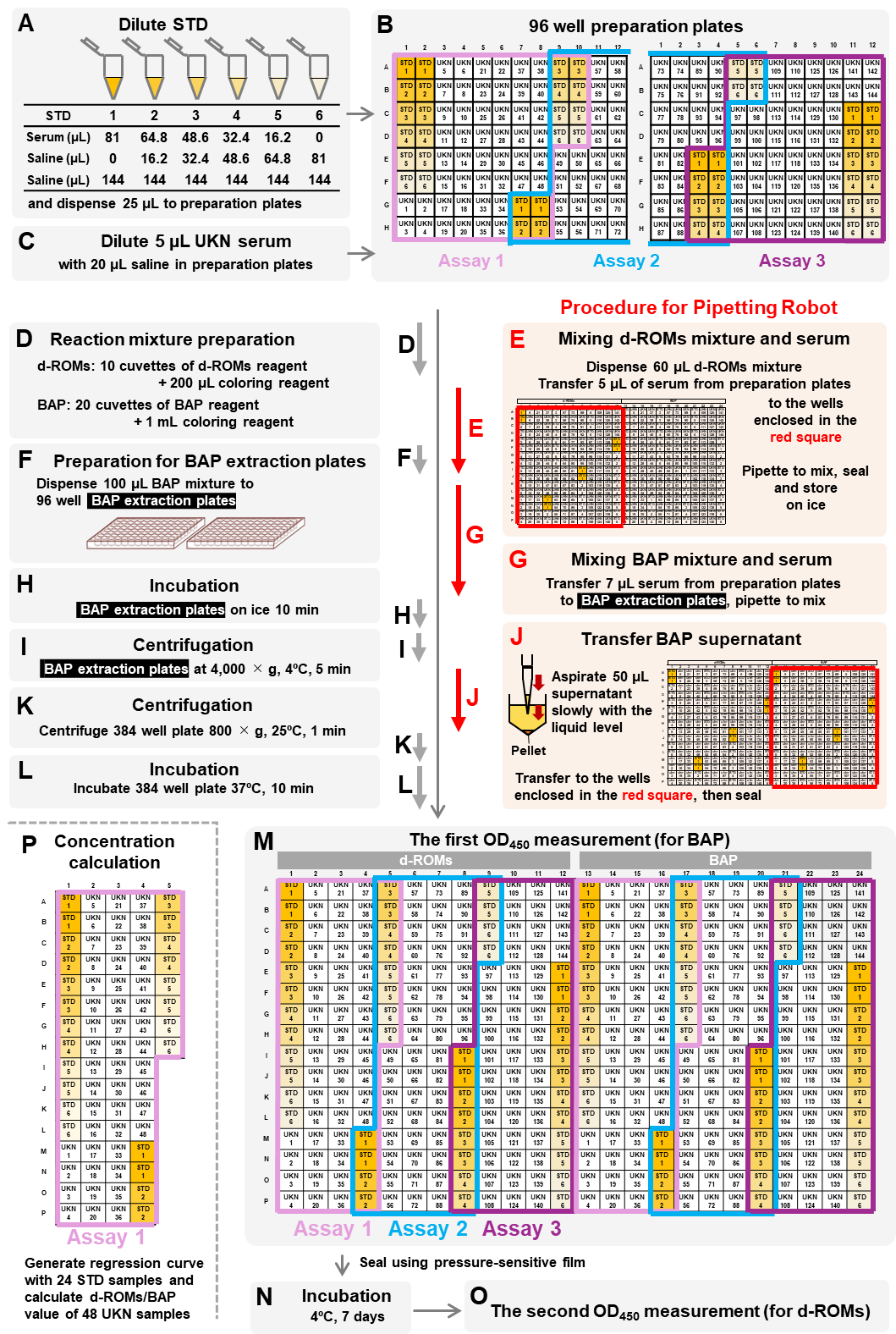
High-Throughput, low volume d-ROMs and BAP Assays: 384-Well Plate Method for Large-Scale Studies**

**Figure S1: Procedure for small-scale d-ROMs and BAP measurement.** Step A to P outlines our procedure for simultaneously measuring d-ROMs and BAP for up to 144 unknown serum samples (UKN).

**(A) Preparation of Standard Serum (STD):** Pooled serum was diluted (2.8- to 13.9-fold) with physiological saline (Otsuka Pharmaceutical, Tokyo, Japan).

**(B) Layout of Preparation Plates:** The standard serum dilutions were dispensed into the wells highlighted in yellow.

**(C) Dilution of UKN:** A 5.0 µL volume of unknown serum (UKN) was mixed with 20 µL of physiological saline, resulting in a 5-fold dilution. This diluted serum was then dispensed into the wells highlighted in white on the 96-well preparation plates (B).

**(D) Reagent Preparation:** The reaction mixtures were prepared by combining either 10 cuvettes of d-ROMs reagent with 200 µL of coloring reagent, or 20 cuvettes of BAP reagent with 1.0 mL of coloring reagent (Wismerll, Tokyo, Japan).

**(E) d-ROMs Assay Set-up:** An Andrew+ pipetting robot (Waters, Milford, MA, USA) dispensed 60 µL of the d-ROMs mixture into the 192 wells on the left half of a 384-well plate (PerkinElmer, Waltham, MA, USA). Then, 5.0 µL of the diluted serum from the 96-well preparation plates was transferred to the same 384-well plate. The plate was sealed and temporarily stored on ice.

**(F, G, H, I) BAP Assay Processing:** The BAP mixture (100 µL) was manually dispensed into a separate set of 96-well BAP extraction plates. The pipetting robot transferred 7.0 µL of the diluted serum from the 96-well preparation plates to the BAP extraction plates. The plate was sealed, incubated on ice for 10 minutes, and then centrifuged at 4,000 × g at 4ºC for five minutes to remove precipitates that could interfere with the measurement.

**(J) Supernatant Transfer:** Using the pipetting robot, 50 µL of the supernatant from the BAP extraction plates was transferred to the vacant wells of the 384-well plate. The robotic protocol was optimized to avoid disturbing the pellet. The aspiration speed was set to “slow,” and the tip position was adjusted "with respect to liquid" (liquid level tracking).

**(K, L, M) BAP Measurement:** The 384-well plate was resealed, centrifuged at 800 × g at 25ºC for one minute, and incubated at 37ºC for 10 minutes. OD_450_ was then measured by a Multiskan FC plate reader (Thermo Fisher Scientific, Waltham, MA, USA) for BAP.

**(N, O) d-ROMs Measurement:** The same 384-well plate was resealed using a pressure-sensitive adhesive film and incubated at 4ºC for seven days. The OD_450_ value was measured again for the d-ROMs analysis.

**(P) Calculation:** A standard curve based on 24 STDs was used for concentration calculation.


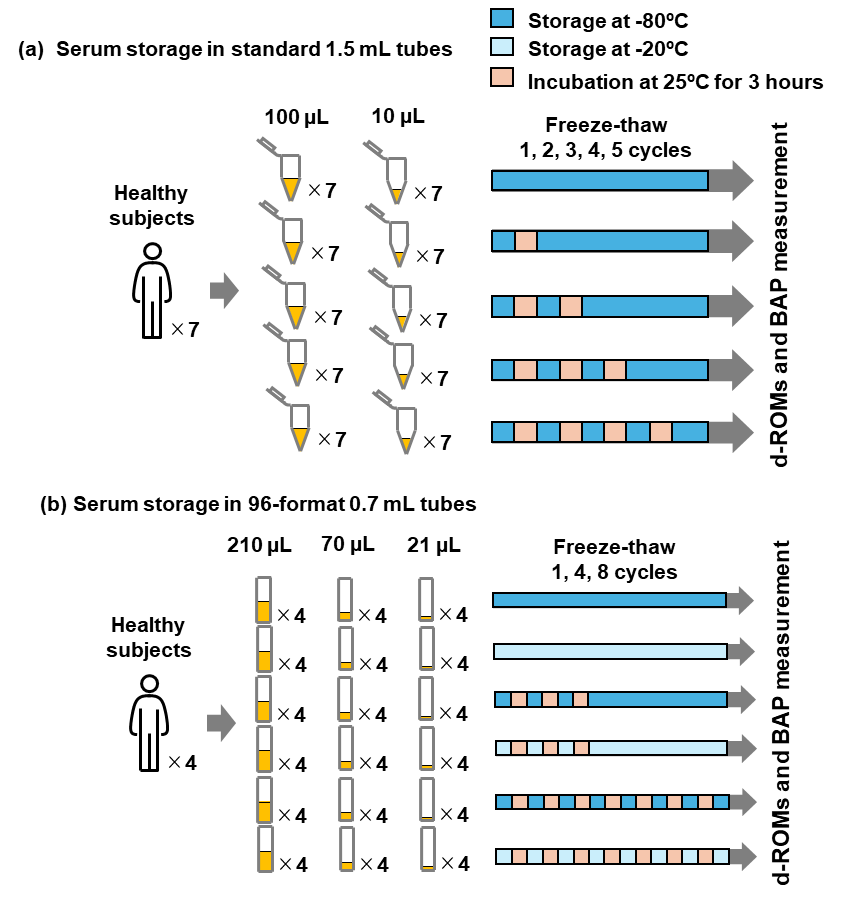


**Figure S2. Freezing and thawing of serum for sample stability test.**

(a) Seven normal sera were obtained from seven healthy individuals. Each serum sample was aliquoted into five standard 1.5 mL tubes (100 µL or 10 µL each) and subjected to one to five freeze-thaw cycles between −80℃ and 25℃.

(b) Four normal sera were obtained from four healthy individuals. Each serum sample was aliquoted into six 96-format 0.7 mL tubes (210 µL, 70 µL, or 21 µL each) and subjected to one to eight freeze-thaw cycles between −80℃ or −20℃ and 25℃.**Table S1. Reproducibility of lipemic serum.**

| Serum E | d-ROMs = 378 U.CARR* | | | | | | | | |  | BAP = 978 µM* | | | | |
| --- | --- | --- | --- | --- | --- | --- | --- | --- | --- | --- | --- | --- | --- | --- | --- |
|  | 10 min at 37ºC | | | |  | 7 days at 4ºC | | | |  | 10 min at 37ºC | | | | |
| Assay number | 1 | 2 | 3 | 1–3 |  | 4 | 5 | 6 | 4–6 |  | 1 | 2 | 3 | 1–3 |  |
|  | (4) | (4) | (4) | (12) |  | (4) | (4) | (4) | (12) |  | (4) | (4) | (4) | (12) |  |
| Mean | 692 | 750 | 693 | 712 |  | 412 | 433 | 411 | 419 |  | 1905 | 1756 | 1868 | 1843 |  |
| CV | 3% | 4% | 6% | 6% |  | 5% | 4% | 2% | 4% |  | 8% | 6% | 9% | 8% |  |
| Δ** | 83% | 98% | 83% | 88% |  | 9% | 15% | 9% | 11% |  | 95% | 80% | 91% | 88% |  |
|  |  |  |  |  |  |  |  |  |  |  |  |  |  |  |  |
| Serum F | d-ROMs = 249 U.CARR* | | | | | | | | |  | BAP = 758 µM* | | | | |
|  | 10 min at 37ºC | | | |  | 7 days at 4ºC | | | |  | 10 min at 37ºC | | | | |
| Assay number | 1 | 2 | 3 | 1–3 |  | 4 | 5 | 6 | 4–6 |  | 1 | 2 | 3 | 1–3 |  |
|  | (4) | (4) | (4) | (12) |  | (4) | (4) | (4) | (12) |  | (4) | (4) | (4) | (12) |  |
| Mean | 575 | 474 | 559 | 536 |  | 286 | 282 | 299 | 289 |  | 1770 | 1811 | 1670 | 1751 |  |
| CV | 10% | 4% | 4% | 11% |  | 1% | 10% | 3% | 6% |  | 3% | 5% | 6% | 6% |  |
| Δ** | 131% | 91% | 125% | 115% |  | 15% | 13% | 20% | 16% |  | 134% | 139% | 120% | 131% |  |

d-ROMs and BAP of lipemic serum E and F were performed. The number in brackets indicates the number of repetitions. *Obtained using the conventional method (FREE Carrio Duo). ** Difference from FREE Carrio Duo.

| **Table S2. Calibration stability.** | | | | | | | | | | | | | | | | | | | |
| --- | --- | --- | --- | --- | --- | --- | --- | --- | --- | --- | --- | --- | --- | --- | --- | --- | --- | --- | --- |
| 10 min at 37 ºC | Day 1 | | | | |  | Day 2 | | | | |  | Day 3 | | | | |  | Day 1–3 |
|  | STD Series | | | |  |  | STD Series | | | |  |  | STD Series | | | |  |  |  |
|  | First | Second | Third | Fourth | CV |  | First | Second | Third | Fourth | CV |  | First | Second | Third | Fourth | CV |  | CV |
| STD1 | 0.278 | 0.254 | 0.237 | 0.233 | 7% |  | 0.248 | 0.231 | 0.207 | 0.211 | 7% |  | 0.296 | 0.27 | 0.258 | 0.259 | 6% |  | 10% |
| (575 U.CARR) | 0.264 | 0.254 | 0.231 | 0.23 |  |  | 0.241 | 0.227 | 0.21 | 0.205 |  |  | 0.291 | 0.271 | 0.254 | 0.256 |  |  |  |
| STD2 | 0.216 | 0.21 | 0.195 | 0.193 | 6% |  | 0.191 | 0.187 | 0.178 | 0.166 | 6% |  | 0.232 | 0.225 | 0.21 | 0.208 | 5% |  | 10% |
| (460 U.CARR) | 0.213 | 0.218 | 0.194 | 0.192 |  |  | 0.187 | 0.192 | 0.174 | 0.164 |  |  | 0.228 | 0.223 | 0.215 | 0.205 |  |  |  |
| STD3 | 0.175 | 0.169 | 0.16 | 0.157 | 5% |  | 0.155 | 0.149 | 0.142 | 0.136 | 5% |  | 0.179 | 0.177 | 0.172 | 0.165 | 3% |  | 9% |
| (345 U.CARR) | 0.17 | 0.165 | 0.156 | 0.154 |  |  | 0.147 | 0.141 | 0.143 | 0.132 |  |  | 0.178 | 0.171 | 0.168 | 0.165 |  |  |  |
| STD4 | 0.132 | 0.129 | 0.119 | 0.119 | 4% |  | 0.117 | 0.109 | 0.11 | 0.109 | 4% |  | 0.137 | 0.129 | 0.125 | 0.126 | 3% |  | 7% |
| (230 U.CARR) | 0.129 | 0.125 | 0.121 | 0.123 |  |  | 0.119 | 0.108 | 0.113 | 0.11 |  |  | 0.133 | 0.131 | 0.131 | 0.126 |  |  |  |
| STD5 | 0.094 | 0.092 | 0.091 | 0.091 | 2% |  | 0.086 | 0.081 | 0.081 | 0.082 | 3% |  | 0.091 | 0.09 | 0.09 | 0.094 | 3% |  | 5% |
| (115 U.CARR) | 0.093 | 0.09 | 0.087 | 0.091 |  |  | 0.083 | 0.081 | 0.079 | 0.084 |  |  | 0.089 | 0.087 | 0.089 | 0.095 |  |  |  |
| STD6 | 0.059 | 0.062 | 0.064 | 0.066 | 5% |  | 0.056 | 0.058 | 0.061 | 0.058 | 5% |  | 0.06 | 0.057 | 0.061 | 0.062 | 6% |  | 6% |
| (0 U.CARR) | 0.058 | 0.06 | 0.063 | 0.067 |  |  | 0.055 | 0.057 | 0.062 | 0.063 |  |  | 0.056 | 0.065 | 0.059 | 0.065 |  |  |  |
|  |  |  |  |  |  |  |  |  |  |  |  |  |  |  |  |  |  |  |  |
| 7 days at 4ºC | Day 1 | | | | |  | Day 2 | | | | |  | Day 3 | | | | |  | Day 1–3 |
|  | STD Series | | | |  |  | STD Series | | | |  |  | STD Series | | | |  |  |  |
| U.CARR | First | Second | Third | Fourth | CV |  | First | Second | Third | Fourth | CV |  | First | Second | Third | Fourth | CV |  | CV |
| STD1 | 1.739 | 1.705 | 1.697 | 1.642 | 2% |  | 1.698 | 1.673 | 1.652 | 1.652 | 1% |  | 1.529 | 1.743 | 1.739 | 1.718 | 4% |  | 3% |
| (575 U.CARR) | 1.706 | 1.707 | 1.708 | 1.633 |  |  | 1.679 | 1.668 | 1.686 | 1.649 |  |  | 1.725 | 1.745 | 1.721 | 1.731 |  |  |  |
| STD2 | 1.47 | 1.451 | 1.481 | 1.4 | 3% |  | 1.421 | 1.454 | 1.446 | 1.407 | 2% |  | 1.467 | 1.534 | 1.49 | 1.469 | 3% |  | 3% |
| (460 U.CARR) | 1.489 | 1.549 | 1.454 | 1.43 |  |  | 1.42 | 1.497 | 1.445 | 1.424 |  |  | 1.457 | 1.36 | 1.506 | 1.471 |  |  |  |
| STD3 | 1.291 | 1.219 | 1.222 | 1.219 | 5% |  | 1.201 | 1.172 | 1.219 | 1.209 | 2% |  | 1.225 | 1.218 | 1.26 | 1.223 | 2% |  | 4% |
| (345 U.CARR) | 1.355 | 1.166 | 1.197 | 1.2 |  |  | 1.18 | 1.142 | 1.235 | 1.19 |  |  | 1.244 | 1.175 | 1.271 | 1.22 |  |  |  |
| STD4 | 1.036 | 0.9 | 0.933 | 0.916 | 5% |  | 0.941 | 0.883 | 0.954 | 0.943 | 4% |  | 1.002 | 0.897 | 0.977 | 0.952 | 5% |  | 5% |
| (230 U.CARR) | 0.995 | 0.897 | 0.961 | 0.921 |  |  | 0.94 | 0.893 | 0.994 | 0.943 |  |  | 0.989 | 0.909 | 1.042 | 0.958 |  |  |  |
| STD5 | 0.675 | 0.572 | 0.594 | 0.577 | 11% |  | 0.595 | 0.558 | 0.567 | 0.599 | 4% |  | 0.626 | 0.559 | 0.539 | 0.616 | 6% |  | 7% |
| (115 U.CARR) | 0.741 | 0.571 | 0.559 | 0.581 |  |  | 0.602 | 0.561 | 0.56 | 0.603 |  |  | 0.631 | 0.564 | 0.597 | 0.624 |  |  |  |
| STD6 | 0.199 | 0.14 | 0.148 | 0.183 | 21% |  | 0.121 | 0.124 | 0.145 | 0.136 | 14% |  | 0.122 | 0.113 | 0.14 | 0.172 | 23% |  | 20% |
| (0 U.CARR) | 0.132 | 0.136 | 0.146 | 0.099 |  |  | 0.116 | 0.125 | 0.151 | 0.095 |  |  | 0.119 | 0.116 | 0.195 | 0.108 |  |  |  |
|  |  |  |  |  |  |  |  |  |  |  |  |  |  |  |  |  |  |  |  |
| 10 min at 37 ºC | Day 1 | | | | |  | Day 2 | | | | |  | Day 3 | | | | |  | Day 1–3 |
|  | STD Series | | | |  |  | STD Series | | | |  |  | STD Series | | | |  |  |  |
|  | First | Second | Third | Fourth | CV |  | First | Second | Third | Fourth | CV |  | First | Second | Third | Fourth | CV |  | CV |
| STD1 | 0.2 | 0.186 | 0.179 | 0.174 | 6% |  | 0.224 | 0.225 | 0.217 | 0.207 | 3% |  | 0.216 | 0.216 | 0.209 | 0.192 | 5% |  | 8% |
| (5410 µM ) | 0.189 | 0.2 | 0.175 | 0.178 |  |  | 0.215 | 0.219 | 0.215 | 0.209 |  |  | 0.214 | 0.217 | 0.206 | 0.197 |  |  |  |
| STD2 | 0.221 | 0.211 | 0.205 | 0.206 | 4% |  | 0.265 | 0.26 | 0.264 | 0.256 | 3% |  | 0.257 | 0.253 | 0.254 | 0.239 | 3% |  | 10% |
| (4328 µM) | 0.22 | 0.203 | 0.206 | 0.201 |  |  | 0.268 | 0.252 | 0.281 | 0.26 |  |  | 0.261 | 0.248 | 0.254 | 0.242 |  |  |  |
| STD3 | 0.267 | 0.261 | 0.256 | 0.253 | 2% |  | 0.313 | 0.307 | 0.315 | 0.304 | 1% |  | 0.307 | 0.298 | 0.306 | 0.294 | 2% |  | 8% |
| (3246 µM) | 0.267 | 0.259 | 0.253 | 0.252 |  |  | 0.31 | 0.308 | 0.31 | 0.309 |  |  | 0.307 | 0.295 | 0.305 | 0.295 |  |  |  |
| STD4 | 0.316 | 0.323 | 0.307 | 0.313 | 3% |  | 0.356 | 0.362 | 0.358 | 0.358 | 1% |  | 0.35 | 0.346 | 0.346 | 0.35 | 1% |  | 6% |
| (2164 µM) | 0.322 | 0.327 | 0.304 | 0.311 |  |  | 0.359 | 0.361 | 0.351 | 0.359 |  |  | 0.354 | 0.351 | 0.343 | 0.347 |  |  |  |
| STD5 | 0.371 | 0.375 | 0.369 | 0.366 | 1% |  | 0.405 | 0.406 | 0.404 | 0.403 | 1% |  | 0.399 | 0.395 | 0.383 | 0.385 | 3% |  | 4% |
| (1082 µM) | 0.369 | 0.376 | 0.366 | 0.365 |  |  | 0.404 | 0.411 | 0.398 | 0.406 |  |  | 0.4 | 0.397 | 0.368 | 0.381 |  |  |  |
| STD6 | 0.419 | 0.428 | 0.418 | 0.416 | 1% |  | 0.446 | 0.445 | 0.444 | 0.441 | 1% |  | 0.435 | 0.442 | 0.433 | 0.421 | 2% |  | 3% |
| (0 µM) | 0.421 | 0.429 | 0.424 | 0.414 |  |  | 0.442 | 0.445 | 0.443 | 0.437 |  |  | 0.441 | 0.442 | 0.439 | 0.42 |  |  |  |
| OD_450_ of four standard series was measured on three discontinuous days. | | | | | | | | | | | | | | | | | | | |

**Table S3. Reproducibility of hemolyzed serum.**

| Serum G | d-ROMs = 277 U.CARR* | | | | | | | | |  | BAP = 2047 µM* | | | | |
| --- | --- | --- | --- | --- | --- | --- | --- | --- | --- | --- | --- | --- | --- | --- | --- |
|  | 10 min at 37ºC | | | |  | 7 days at 4ºC | | | |  | 10 min at 37ºC | | | | |
| Assay number | 1 | 2 | 3 | 1–3 |  | 4 | 5 | 6 | 4–6 |  | 1 | 2 | 3 | 1–3 |  |
|  | (4) | (4) | (4) | (12) |  | (4) | (4) | (4) | (12) |  | (4) | (4) | (4) | (12) |  |
| Mean | 314 | 291 | 312 | 306 |  | 296 | 281 | 271 | 283 |  | 1897 | 1904 | 2315 | 2039 |  |
| CV | 1% | 0% | 2% | 4% |  | 2% | 1% | 1% | 4% |  | 1% | 2% | 13% | 13% |  |
| Δ** | 13% | 5% | 13% | 10% |  | 7% | 2% | -2% | 2% |  | -7% | -7% | 13% | 0% |  |
|  |  |  |  |  |  |  |  |  |  |  |  |  |  |  |  |
| Serum H | d-ROMs = 283 U.CARR* | | | | | | | | |  | BAP = 2442 µM* | | | | |
|  | 10 min at 37ºC | | | |  | 7 days at 4ºC | | | |  | 10 min at 37ºC | | | | |
| Assay number | 1 | 2 | 3 | 1–3 |  | 4 | 5 | 6 | 4–6 |  | 1 | 2 | 3 | 1–3 |  |
|  | (4) | (4) | (4) | (12) |  | (4) | (4) | (4) | (12) |  | (4) | (4) | (4) | (12) |  |
| Mean | 306 | 281 | 289 | 292 |  | 310 | 288 | 272 | 290 |  | 2358 | 2195 | 2561 | 2371 |  |
| CV | 1% | 2% | 4% | 4% |  | 6% | 2% | 2% | 7% |  | 2% | 4% | 7% | 8% |  |
| Δ** | 8% | -1% | 2% | 3% |  | 10% | 2% | -4% | 2% |  | -3% | -10% | 5% | -3% |  |

d-ROMs and BAP of hemolyzed serum G and H were performed. The number in brackets indicates the number of repetitions. *Obtained using the conventional method (FREE Carrio Duo). ** Difference from FREE Carrio Duo.

| **Table S4. Recovery rate.** | | | | | |
| --- | --- | --- | --- | --- | --- |
|  | Dilution  factor | Expected value | Mean (n = 4) | Coefficient of Variance | Recovery |
| d-ROMs = 383 U.CARR* | | | | | |
|  | 1 | 383 | 408 | 1% | 106% |
|  | 0.8 | 306 | 318 | 3% | 104% |
|  | 0.6 | 230 | 260 | 2% | 113% |
|  | 0.4 | 153 | 155 | 3% | 101% |
|  | 0.2 | 77 | 65 | 7% | 85% |
| BAP = 2263 μM* | | | | | |
|  | 1 | 2263 | 2024 | 10% | 89% |
|  | 0.8 | 1810 | 1887 | 7% | 104% |
|  | 0.6 | 1358 | 1253 | 12% | 92% |
|  | 0.4 | 905 | 992 | 10% | 110% |
|  | 0.2 | 453 | 472 | 15% | 104% |
| The recovery rate was examined for a five-level serum dilution series as the ratio of the measured values to the expected values. *Obtained using the conventional method (FREE Carrio Duo). | | | | | |

**Figure S3. Intra-plate variations in 384-well microplate assays.** A serum was dispensed into all the wells of a 384-well plate. Colorimetric measurements were performed on d-ROMs in the left half of the plate (192 wells) and on BAP in the right half (192 wells) using the current method, and the absorbances were plotted. (a) Dot plots showing the distribution of absorbance at 450 nm across columns 1–12 for d-ROMs and 13–24 for BAP. Error bars represent the median ± interquartile range (IQR) for each column. Arrows represent the column range of each assay. The percentages show the range in the median value of coefficients of variation (CV) for corresponding columns (16 wells each) within the column ranges. (b) Dot plots showing the distribution of absorbance at 450 nm across rows A–P. Horizontal bars indicate the median ± IQR for each row. (c) Heatmaps visualize intra-plate variations for d-ROMs (top) and BAP (bottom). The left panel displays the combined data from assays 1 through 3. The right panel shows a heat map of all the wells used in an individual assay run, along with their respective CV. Wells not included in the assay are indicated by an "x."

**Table S5. Reproducibility of normal serum.**

|  |  | Day 1 | | | |  | Day 2 | | | |  | Day 3 | | | |  | Day 1–3 |
| --- | --- | --- | --- | --- | --- | --- | --- | --- | --- | --- | --- | --- | --- | --- | --- | --- | --- |
|  |  | Intra assay | | | Inter assay |  | Intra assay | | | Inter assay |  | Intra assay | | | Inter assay |  | Inter day |
| Assay number | | 1 | 2 | 3 | 1–3 |  | 4 | 5 | 6 | 4–6 |  | 7 | 8 | 9 | 7–9 |  | 1–9 |
| Serum A | d-ROMs = 204 U.CARR* | | | | |  |  |  |  |  |  |  |  |  |  |  |  |
|  |  | (16) | (16) | (16) | (48) |  | (16) | (16) | (16) | (48) |  | (12) | (12) | (12) | (36) |  | (132) |
|  | Mean | 198 | 208 | 204 | 204 |  | 214 | 211 | 206 | 210 |  | 222 | 216 | 210 | 216 |  | 209 |
|  | CV | 7% | 5% | 4% | 6% |  | 7% | 4% | 4% | 5% |  | 9% | 6% | 4% | 7% |  | 6% |
|  | Δ** | -3% | 2% | 0% | 0% |  | 5% | 3% | 1% | 3% |  | 9% | 6% | 3% | 6% |  | 3% |
| Serum B | d-ROMs = 486 U.CARR* | | | | |  |  |  |  |  |  |  |  |  |  |  |  |
|  |  | (16) | (16) | (16) | (48) |  | (16) | (16) | (16) | (48) |  | (12) | (12) | (12) | (36) |  | (132) |
|  | Mean | 457 | 444 | 452 | 451 |  | 462 | 464 | 476 | 467 |  | 456 | 471 | 482 | 470 |  | 462 |
|  | CV | 7% | 5% | 5% | 5% |  | 6% | 5% | 5% | 5% |  | 8% | 6% | 6% | 7% |  | 6% |
|  | Δ** | -6% | -9% | -7% | -7% |  | -5% | -5% | -2% | -4% |  | -6% | -3% | -1% | -3% |  | -5% |
| Serum C | d-ROMs = 172 U.CARR* | | | | |  |  |  |  |  |  |  |  |  |  |  |  |
|  |  | (4) | (4) | (4) | (12) |  | (4) | (4) | (4) | (12) |  | (4) | (4) | (4) | (12) |  | (36) |
|  | Mean | 158 | 171 | 159 | 163 |  | 184 | 169 | 156 | 170 |  | 176 | 176 | 166 | 173 |  | 168 |
|  | CV | 6% | 4% | 4% | 6% |  | 2% | 1% | 2% | 7% |  | 2% | 3% | 8% | 9% |  | 6% |
|  | Δ** | -8% | 0% | -7% | -5% |  | 7% | -1% | -9% | -1% |  | 3% | 2% | -3% | 1% |  | -2% |
| Serum D | d-ROMs = 176 U.CARR* | | | | |  |  |  |  |  |  |  |  |  |  |  |  |
|  |  | (4) | (4) | (4) | (12) |  | (4) | (4) | (4) | (12) |  | (4) | (4) | (4) | (12) |  | (36) |
|  | Mean | 154 | 181 | 176 | 170 |  | 168 | 184 | 171 | 175 |  | 202 | 186 | 171 | 186 |  | 177 |
|  | CV | 4% | 2% | 3% | 8% |  | 4% | 3% | 2% | 5% |  | 4% | 4% | 1% | 8% |  | 8% |
|  | Δ** | -13% | 3% | 0% | -3% |  | -4% | 5% | -3% | -1% |  | 15% | 6% | -3% | 6% |  | 1% |
| Serum A | BAP = 2700 µM* | | |  |  |  |  |  |  |  |  |  |  |  |  |  |  |
|  |  | (16) | (16) | (16) | (48) |  | (16) | (16) | (16) | (48) |  | (12) | (12) | (12) | (36) |  | (132) |
|  | Mean | 2422 | 2388 | 2421 | 2410 |  | 2482 | 2478 | 2500 | 2487 |  | 2507 | 2459 | 2451 | 2472 |  | 2455 |
|  | CV | 3% | 3% | 2% | 3% |  | 3% | 3% | 3% | 3% |  | 3% | 4% | 4% | 4% |  | 3% |
|  | Δ** | -10% | -12% | -10% | -11% |  | -8% | -8% | -7% | -8% |  | -7% | -9% | -9% | -8% |  | -9% |
| Serum B | BAP = 3485 µM* | | |  |  |  |  |  |  |  |  |  |  |  |  |  |  |
|  |  | (16) | (16) | (16) | (48) |  | (16) | (16) | (16) | (48) |  | (12) | (12) | (12) | (36) |  | (132) |
|  | Mean | 3067 | 3017 | 3161 | 3082 |  | 3199 | 3218 | 3208 | 3209 |  | 3281 | 3292 | 3359 | 3311 |  | 3190 |
|  | CV | 4% | 7% | 2% | 5% |  | 2% | 3% | 3% | 3% |  | 2% | 3% | 5% | 4% |  | 5% |
|  | Δ** | -12% | -13% | -9% | -12% |  | -8% | -8% | -8% | -8% |  | -6% | -6% | -4% | -5% |  | -8% |
| Serum C | BAP = 1211 µM* | | |  |  |  |  |  |  |  |  |  |  |  |  |  |  |
|  |  | (4) | (4) | (4) | (12) |  | (4) | (4) | (4) | (12) |  | (4) | (4) | (4) | (12) |  | (36) |
|  | Mean | 1167 | 1021 | 1015 | 1067 |  | 1188 | 1052 | 975 | 1072 |  | 1059 | 1042 | 1115 | 1072 |  | 1070 |
|  | CV | 8% | 6% | 4% | 9% |  | 12% | 3% | 2% | 11% |  | 3% | 4% | 17% | 11% |  | 10% |
|  | Δ** | -4% | -16% | -16% | -12% |  | -2% | -13% | -19% | -11% |  | -13% | -14% | -8% | -11% |  | -12% |
| Serum D | BAP = 1467 µM* | | |  |  |  |  |  |  |  |  |  |  |  |  |  |  |
|  |  | (4) | (4) | (4) | (12) |  | (4) | (4) | (4) | (12) |  | (4) | (4) | (4) | (12) |  | (36) |
|  | Mean | 1310 | 1316 | 1280 | 1302 |  | 1355 | 1346 | 1177 | 1293 |  | 1363 | 1191 | 1386 | 1314 |  | 1303 |
|  | CV | 5% | 3% | 7% | 5% |  | 3% | 6% | 3% | 8% |  | 6% | 2% | 9% | 9% |  | 7% |
|  | Δ** | -11% | -10% | -13% | -11% |  | -8% | -8% | -20% | -12% |  | -7% | -19% | -5% | -10% |  | -11% |

d-ROMs and BAP of serum A to D were performed for 3 discontinuous days. The number in brackets indicates the number of repetitions. *Obtained using the conventional method (FREE Carrio Duo). ** Difference from FREE Carrio Duo.

| **Table S6. Inter-operator variation assay.** | |  |  |  |  |
| --- | --- | --- | --- | --- | --- |
|  |  | Intra operator | | | Inter operator |
|  | | Operator 1 | Operator 2 | Operator 3 | Operator 1-3 |
| Serum A | d-ROMs = 383 U.CARR* | (4) | (4) | (4) | (12) |
|  | Mean | 403 | 408 | 431 | 414 |
|  | CV | 3% | 1% | 3% | 4% |
| Serum B | d-ROMs = 296 U.CARR* | (4) | (4) | (4) | (12) |
|  | Mean | 215 | 245 | 224 | 228 |
|  | CV | 4% | 2% | 3% | 6% |
| Serum C | d-ROMs = 237 U.CARR* | (4) | (4) | (4) | (12) |
|  | Mean | 171 | 189 | 198 | 186 |
|  | CV | 2% | 2% | 1% | 7% |
| Serum A | BAP = 2263 μM* | (4) | (4) | (4) | (12) |
|  | Mean | 2333 | 2024 | 2171 | 2176 |
|  | CV | 5% | 10% | 7% | 9% |
| Serum B | BAP = 1955 μM* | (4) | (4) | (4) | (12) |
|  | Mean | 1967 | 2180 | 2135 | 2094 |
|  | CV | 9% | 6% | 5% | 8% |
| Serum C | BAP = 1564 μM* | (4) | (4) | (4) | (12) |
|  | Mean | 1623 | 1809 | 1598 | 1677 |
|  | CV | 6% | 5% | 7% | 8% |
| d-ROMs and BAP of serum A to C were measured by three operators. The number in brackets indicates the number of repetitions. *Obtained using the conventional system (FREE Cariio Duo). | | | | | |

| **Table S7. Effects of storage conditions.** | |  |  |
| --- | --- | --- | --- |
| **Storage in standard 1.5 mL tubes** | |  |  |
| ln(d-ROMs, 144 observations) | |  |  |
|  | Variables | β | p-value |
|  | Storage volume (10 μL vs 100 μL) | 0.138 | 0.004 |
|  | Freeze-thaw cycle (per cycle) | −0.003 | 0.804 |
|  | Storage volume × freeze-thaw cycle | −0.003 | 0.804 |
| ln(BAP, 144 observations) | |  |  |
|  | Variables | β | p-value |
|  | Storage volume (10 μL vs 100 μL) | −0.056 | 0.326 |
|  | Freeze-thaw cycle (per cycle) | 0.001 | 0.948 |
|  | Storage volume × freeze-thaw cycle | 0.038 | 0.016 |
| **Storage in 96-format 0.7 mL tubes** | |  |  |
| ln(d-ROMs) | |  |  |
|  | All (144 observations) |  |  |
|  | Variables | β | p-value |
|  | Storage volume (order, 1 = 21 μL, 2 = 70 μL, 3 = 210 μL) | 0.012 | 0.106 |
|  | Freeze-thaw cycle (order, 1 = once, 2 = fourth, 3 = eighth) | 0.004 | 0.587 |
|  | Storage at −20ºC (vs −80ºC) | -0.002 | <.0001 |
|  |  |  |  |
|  | Storage at -80ºC (72 observations) |  |  |
|  | Variables | β | p-value |
|  | Storage volume (order, 1 = 21 μL, 2 = 70 μL, 3 = 210 μL) | 0.021 | 0.357 |
|  | Freeze-thaw cycle (order, 1 = once, 2 = fourth, 3 = eighth) | 0.034 | 0.146 |
|  | Storage volume × freeze-thaw cycle | -0.013 | 0.241 |
|  | Storage at -20ºC (72 observations) |  |  |
|  | Variables | β | p-value |
|  | Storage volume (1 = 21 μL, 2 = 70 μL, 3 = 210 μL) | -0.033 | 0.234 |
|  | Freeze-thaw cycle (1 = once, 2 = fourth, 3 = eighth) | -0.061 | 0.031 |
|  | Storage volume × freeze-thaw cycle | 0.030 | 0.023 |
| ln(BAP) | |  |  |
|  | All (144 observations) |  |  |
|  | Variables | β | p-value |
|  | Storage volume (order, 1 = 21 μL, 2 = 70 μL, 3 = 210 μL) | 0.008 | 0.482 |
|  | Freeze-thaw cycle (order, 1 = once, 2 = fourth, 3 = eighth) | 0.013 | 0.265 |
|  | Storage at −20ºC (vs −80ºC) | 0.000 | 0.126 |
|  |  |  |  |
|  | Storage at -80ºC (72 observations) |  |  |
|  | Variables | β | p-value |
|  | Storage volume (order, 1 = 21 μL, 2 = 70 μL, 3 = 210 μL) | -0.029 | 0.448 |
|  | Freeze-thaw cycle (order, 1 = once, 2 = fourth, 3 = eighth) | -0.030 | 0.439 |
|  | Storage volume × freeze-thaw cycle | 0.013 | 0.480 |
|  | Storage at -20ºC (72 observations) |  |  |
|  | Variables | β | p-value |
|  | Storage volume (1 = 21 μL, 2 = 70 μL, 3 = 210 μL) | -0.021 | 0.568 |
|  | Freeze-thaw cycle (1 = once, 2 = fourth, 3 = eighth) | -0.011 | 0.764 |
|  | Storage volume × freeze-thaw cycle | 0.020 | 0.232 |
| **Storage effects in standard 1.5 mL tubes** were examined using seven sera from seven individuals (see Figure S2). Each serum sample was divided into ten tubes: five containing 100 µL of serum and five containing 10 µL of serum. A freeze-thaw cycle was repeated one to five times. All serum samples were then thawed and measured in an assay. **Storage effects in 96-format 0.7 mL tubes** were examined using serum from four individuals. Samples were frozen and thawed under 18 different conditions and analysed using the current method (four serum samples × 18 conditions = 72 observations, with duplicate measurements providing a total of 144 observations). Mixed-effects models that allow for random effect for sample specificity were used to examine the effect of these storage conditions on ln(d-ROMs) and ln(BAP) (PROC MIXED, SAS9.4). β, partial regression coefficient. | | | |

**
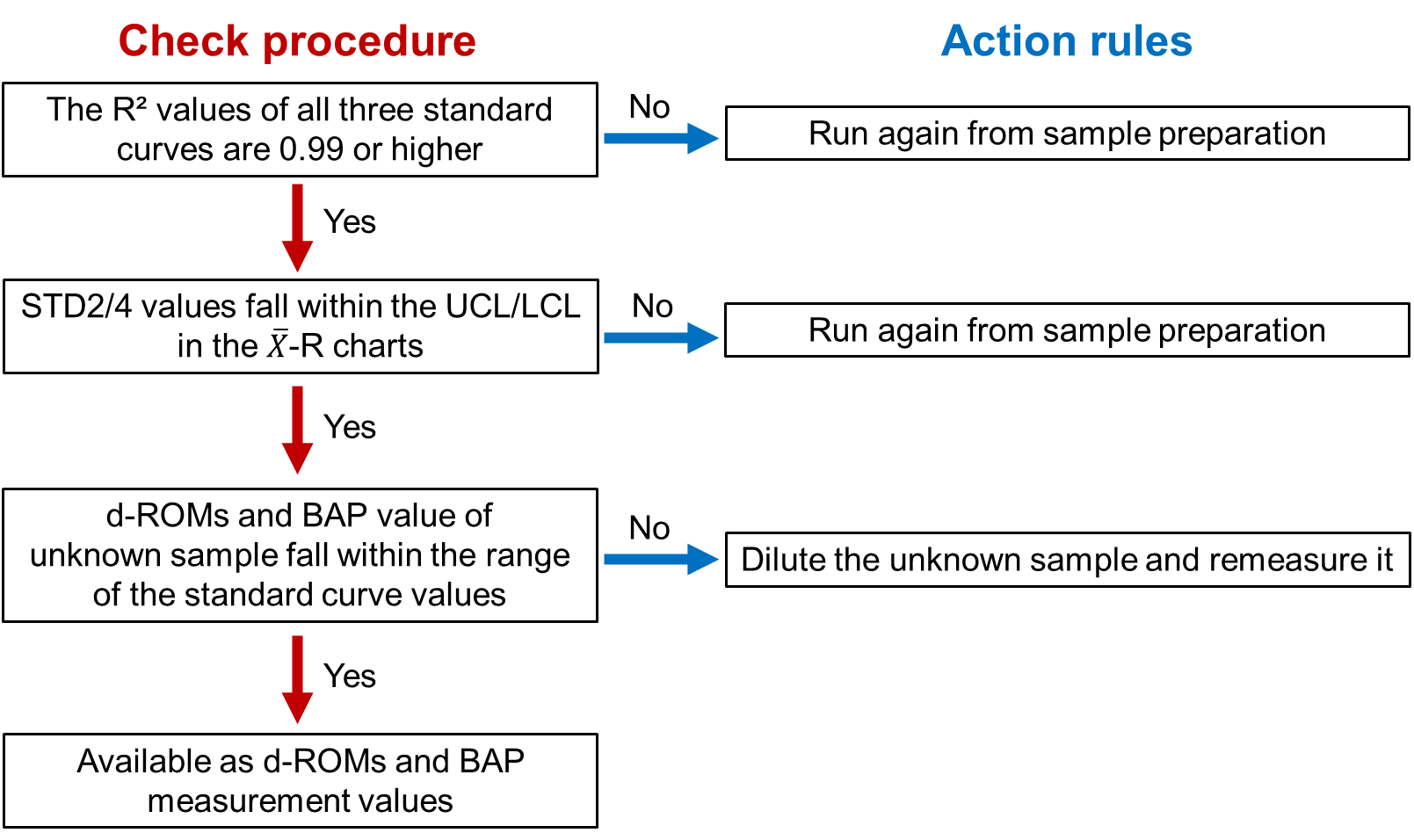
**

**Figure S4. Quality control framework and decision-making flowchart for d-ROMs and BAP assays.**

The integrity of each analytical run was evaluated through a hierarchical three-step verification process. First, the linearity of three independent standard curves must be confirmed (R^2^ ≥ 0.99). Second, the monitored values for STD2 and STD4 must remain within predefined control limits. Third, each unknown sample must fall within the validated linear range of the standard curve. Failure to meet any of these criteria results in a reanalysis as suggested in the figure.
