# Supplementary material for: High-throughput, low volume d-ROMs and BAP assays: 384-well plate method for large-scale studies
Source: Environ Health Prev Med. 2026 Jul 3;31:42. doi: 10.1265/ehpm.25-00354 (PMC13366183; doi:10.1265/ehpm.25-00354)
Supplement: Supplementary file 3 — Additional file 3: Andrew pipetting protocol_BAP-1. [file ehpm-31-042-s003.pdf]

ol-bap-1-template

LabwareName	Volume	SolutionName	SolutionType	ConcentrationValue	ConcentrationUnit	SampleIdentifier	LabwareIdentifier	Step1Source	Step1Volume	Step1Destination
"MicroAmp™ 0.2 mL Fast Optical 96-well se":A1	20	Sample	Reagent	1	a.u.			"MicroAmp™ 0.2 mL Fast Optical 96-well se":A1	7	"MicroAmp™ 0.3 mL optical 96-well semi-sk":A1
"MicroAmp™ 0.2 mL Fast Optical 96-well se":A2	20	Sample	Reagent	1	a.u.			"MicroAmp™ 0.2 mL Fast Optical 96-well se":A2	7	"MicroAmp™ 0.3 mL optical 96-well semi-sk":A2
"MicroAmp™ 0.2 mL Fast Optical 96-well se":A3	20	Sample	Reagent	1	a.u.			"MicroAmp™ 0.2 mL Fast Optical 96-well se":A3	7	"MicroAmp™ 0.3 mL optical 96-well semi-sk":A3
"MicroAmp™ 0.2 mL Fast Optical 96-well se":A4	20	Sample	Reagent	1	a.u.			"MicroAmp™ 0.2 mL Fast Optical 96-well se":A4	7	"MicroAmp™ 0.3 mL optical 96-well semi-sk":A4
"MicroAmp™ 0.2 mL Fast Optical 96-well se":A5	20	Sample	Reagent	1	a.u.			"MicroAmp™ 0.2 mL Fast Optical 96-well se":A5	7	"MicroAmp™ 0.3 mL optical 96-well semi-sk":A5
"MicroAmp™ 0.2 mL Fast Optical 96-well se":A6	20	Sample	Reagent	1	a.u.			"MicroAmp™ 0.2 mL Fast Optical 96-well se":A6	7	"MicroAmp™ 0.3 mL optical 96-well semi-sk":A6
"MicroAmp™ 0.2 mL Fast Optical 96-well se":A7	20	Sample	Reagent	1	a.u.			"MicroAmp™ 0.2 mL Fast Optical 96-well se":A7	7	"MicroAmp™ 0.3 mL optical 96-well semi-sk":A7
"MicroAmp™ 0.2 mL Fast Optical 96-well se":A8	20	Sample	Reagent	1	a.u.			"MicroAmp™ 0.2 mL Fast Optical 96-well se":A8	7	"MicroAmp™ 0.3 mL optical 96-well semi-sk":A8
"MicroAmp™ 0.2 mL Fast Optical 96-well se":A9	20	Sample	Reagent	1	a.u.			"MicroAmp™ 0.2 mL Fast Optical 96-well se":A9	7	"MicroAmp™ 0.3 mL optical 96-well semi-sk":A9
"MicroAmp™ 0.2 mL Fast Optical 96-well se":A10	20	Sample	Reagent	1	a.u.			"MicroAmp™ 0.2 mL Fast Optical 96-well se":A10	7	"MicroAmp™ 0.3 mL optical 96-well semi-sk":A10
"MicroAmp™ 0.2 mL Fast Optical 96-well se":A11	20	Sample	Reagent	1	a.u.			"MicroAmp™ 0.2 mL Fast Optical 96-well se":A11	7	"MicroAmp™ 0.3 mL optical 96-well semi-sk":A11
"MicroAmp™ 0.2 mL Fast Optical 96-well se":A12	20	Sample	Reagent	1	a.u.			"MicroAmp™ 0.2 mL Fast Optical 96-well se":A12	7	"MicroAmp™ 0.3 mL optical 96-well semi-sk":A12
"MicroAmp™ 0.2 mL Fast Optical 96-well se":B1	20	Sample	Reagent	1	a.u.			"MicroAmp™ 0.2 mL Fast Optical 96-well se":B1	7	"MicroAmp™ 0.3 mL optical 96-well semi-sk":B1
"MicroAmp™ 0.2 mL Fast Optical 96-well se":B2	20	Sample	Reagent	1	a.u.			"MicroAmp™ 0.2 mL Fast Optical 96-well se":B2	7	"MicroAmp™ 0.3 mL optical 96-well semi-sk":B2
"MicroAmp™ 0.2 mL Fast Optical 96-well se":B3	20	Sample	Reagent	1	a.u.			"MicroAmp™ 0.2 mL Fast Optical 96-well se":B3	7	"MicroAmp™ 0.3 mL optical 96-well semi-sk":B3
"MicroAmp™ 0.2 mL Fast Optical 96-well se":B4	20	Sample	Reagent	1	a.u.			"MicroAmp™ 0.2 mL Fast Optical 96-well se":B4	7	"MicroAmp™ 0.3 mL optical 96-well semi-sk":B4
"MicroAmp™ 0.2 mL Fast Optical 96-well se":B5	20	Sample	Reagent	1	a.u.			"MicroAmp™ 0.2 mL Fast Optical 96-well se":B5	7	"MicroAmp™ 0.3 mL optical 96-well semi-sk":B5
"MicroAmp™ 0.2 mL Fast Optical 96-well se":B6	20	Sample	Reagent	1	a.u.			"MicroAmp™ 0.2 mL Fast Optical 96-well se":B6	7	"MicroAmp™ 0.3 mL optical 96-well semi-sk":B6
"MicroAmp™ 0.2 mL Fast Optical 96-well se":B7	20	Sample	Reagent	1	a.u.			"MicroAmp™ 0.2 mL Fast Optical 96-well se":B7	7	"MicroAmp™ 0.3 mL optical 96-well semi-sk":B7
"MicroAmp™ 0.2 mL Fast Optical 96-well se":B8	20	Sample	Reagent	1	a.u.			"MicroAmp™ 0.2 mL Fast Optical 96-well se":B8	7	"MicroAmp™ 0.3 mL optical 96-well semi-sk":B8
"MicroAmp™ 0.2 mL Fast Optical 96-well se":B9	20	Sample	Reagent	1	a.u.			"MicroAmp™ 0.2 mL Fast Optical 96-well se":B9	7	"MicroAmp™ 0.3 mL optical 96-well semi-sk":B9
"MicroAmp™ 0.2 mL Fast Optical 96-well se":B10	20	Sample	Reagent	1	a.u.			"MicroAmp™ 0.2 mL Fast Optical 96-well se":B10	7	"MicroAmp™ 0.3 mL optical 96-well semi-sk":B10
"MicroAmp™ 0.2 mL Fast Optical 96-well se":B11	20	Sample	Reagent	1	a.u.			"MicroAmp™ 0.2 mL Fast Optical 96-well se":B11	7	"MicroAmp™ 0.3 mL optical 96-well semi-sk":B11
"MicroAmp™ 0.2 mL Fast Optical 96-well se":B12	20	Sample	Reagent	1	a.u.			"MicroAmp™ 0.2 mL Fast Optical 96-well se":B12	7	"MicroAmp™ 0.3 mL optical 96-well semi-sk":B12
"MicroAmp™ 0.2 mL Fast Optical 96-well se":C1	20	Sample	Reagent	1	a.u.			"MicroAmp™ 0.2 mL Fast Optical 96-well se":C1	7	"MicroAmp™ 0.3 mL optical 96-well semi-sk":C1
"MicroAmp™ 0.2 mL Fast Optical 96-well se":C2	20	Sample	Reagent	1	a.u.			"MicroAmp™ 0.2 mL Fast Optical 96-well se":C2	7	"MicroAmp™ 0.3 mL optical 96-well semi-sk":C2
"MicroAmp™ 0.2 mL Fast Optical 96-well se":C3	20	Sample	Reagent	1	a.u.			"MicroAmp™ 0.2 mL Fast Optical 96-well se":C3	7	"MicroAmp™ 0.3 mL optical 96-well semi-sk":C3
"MicroAmp™ 0.2 mL Fast Optical 96-well se":C4	20	Sample	Reagent	1	a.u.			"MicroAmp™ 0.2 mL Fast Optical 96-well se":C4	7	"MicroAmp™ 0.3 mL optical 96-well semi-sk":C4
"MicroAmp™ 0.2 mL Fast Optical 96-well se":C5	20	Sample	Reagent	1	a.u.			"MicroAmp™ 0.2 mL Fast Optical 96-well se":C5	7	"MicroAmp™ 0.3 mL optical 96-well semi-sk":C5
"MicroAmp™ 0.2 mL Fast Optical 96-well se":C6	20	Sample	Reagent	1	a.u.			"MicroAmp™ 0.2 mL Fast Optical 96-well se":C6	7	"MicroAmp™ 0.3 mL optical 96-well semi-sk":C6
"MicroAmp™ 0.2 mL Fast Optical 96-well se":C7	20	Sample	Reagent	1	a.u.			"MicroAmp™ 0.2 mL Fast Optical 96-well se":C7	7	"MicroAmp™ 0.3 mL optical 96-well semi-sk":C7
"MicroAmp™ 0.2 mL Fast Optical 96-well se":C8	20	Sample	Reagent	1	a.u.			"MicroAmp™ 0.2 mL Fast Optical 96-well se":C8	7	"MicroAmp™ 0.3 mL optical 96-well semi-sk":C8
"MicroAmp™ 0.2 mL Fast Optical 96-well se":C9	20	Sample	Reagent	1	a.u.			"MicroAmp™ 0.2 mL Fast Optical 96-well se":C9	7	"MicroAmp™ 0.3 mL optical 96-well semi-sk":C9
"MicroAmp™ 0.2 mL Fast Optical 96-well se":C10	20	Sample	Reagent	1	a.u.			"MicroAmp™ 0.2 mL Fast Optical 96-well se":C10	7	"MicroAmp™ 0.3 mL optical 96-well semi-sk":C10
"MicroAmp™ 0.2 mL Fast Optical 96-well se":C11	20	Sample	Reagent	1	a.u.			"MicroAmp™ 0.2 mL Fast Optical 96-well se":C11	7	"MicroAmp™ 0.3 mL optical 96-well semi-sk":C11
"MicroAmp™ 0.2 mL Fast Optical 96-well se":C12	20	Sample	Reagent	1	a.u.			"MicroAmp™ 0.2 mL Fast Optical 96-well se":C12	7	"MicroAmp™ 0.3 mL optical 96-well semi-sk":C12
"MicroAmp™ 0.2 mL Fast Optical 96-well se":D1	20	Sample	Reagent	1	a.u.			"MicroAmp™ 0.2 mL Fast Optical 96-well se":D1	7	"MicroAmp™ 0.3 mL optical 96-well semi-sk":D1
"MicroAmp™ 0.2 mL Fast Optical 96-well se":D2	20	Sample	Reagent	1	a.u.			"MicroAmp™ 0.2 mL Fast Optical 96-well se":D2	7	"MicroAmp™ 0.3 mL optical 96-well semi-sk":D2
"MicroAmp™ 0.2 mL Fast Optical 96-well se":D3	20	Sample	Reagent	1	a.u.			"MicroAmp™ 0.2 mL Fast Optical 96-well se":D3	7	"MicroAmp™ 0.3 mL optical 96-well semi-sk":D3
"MicroAmp™ 0.2 mL Fast Optical 96-well se":D4	20	Sample	Reagent	1	a.u.			"MicroAmp™ 0.2 mL Fast Optical 96-well se":D4	7	"MicroAmp™ 0.3 mL optical 96-well semi-sk":D4
"MicroAmp™ 0.2 mL Fast Optical 96-well se":D5	20	Sample	Reagent	1	a.u.			"MicroAmp™ 0.2 mL Fast Optical 96-well se":D5	7	"MicroAmp™ 0.3 mL optical 96-well semi-sk":D5
"MicroAmp™ 0.2 mL Fast Optical 96-well se":D6	20	Sample	Reagent	1	a.u.			"MicroAmp™ 0.2 mL Fast Optical 96-well se":D6	7	"MicroAmp™ 0.3 mL optical 96-well semi-sk":D6
"MicroAmp™ 0.2 mL Fast Optical 96-well se":D7	20	Sample	Reagent	1	a.u.			"MicroAmp™ 0.2 mL Fast Optical 96-well se":D7	7	"MicroAmp™ 0.3 mL optical 96-well semi-sk":D7
"MicroAmp™ 0.2 mL Fast Optical 96-well se":D8	20	Sample	Reagent	1	a.u.			"MicroAmp™ 0.2 mL Fast Optical 96-well se":D8	7	"MicroAmp™ 0.3 mL optical 96-well semi-sk":D8
"MicroAmp™ 0.2 mL Fast Optical 96-well se":D9	20	Sample	Reagent	1	a.u.			"MicroAmp™ 0.2 mL Fast Optical 96-well se":D9	7	"MicroAmp™ 0.3 mL optical 96-well semi-sk":D9
"MicroAmp™ 0.2 mL Fast Optical 96-well se":D10	20	Sample	Reagent	1	a.u.			"MicroAmp™ 0.2 mL Fast Optical 96-well se":D10	7	"MicroAmp™ 0.3 mL optical 96-well semi-sk":D10
"MicroAmp™ 0.2 mL Fast Optical 96-well se":D11	20	Sample	Reagent	1	a.u.			"MicroAmp™ 0.2 mL Fast Optical 96-well se":D11	7	"MicroAmp™ 0.3 mL optical 96-well semi-sk":D11
"MicroAmp™ 0.2 mL Fast Optical 96-well se":D12	20	Sample	Reagent	1	a.u.			"MicroAmp™ 0.2 mL Fast Optical 96-well se":D12	7	"MicroAmp™ 0.3 mL optical 96-well semi-sk":D12
"MicroAmp™ 0.2 mL Fast Optical 96-well se":E1	20	Sample	Reagent	1	a.u.			"MicroAmp™ 0.2 mL Fast Optical 96-well se":E1	7	"MicroAmp™ 0.3 mL optical 96-well semi-sk":E1
"MicroAmp™ 0.2 mL Fast Optical 96-well se":E2	20	Sample	Reagent	1	a.u.			"MicroAmp™ 0.2 mL Fast Optical 96-well se":E2	7	"MicroAmp™ 0.3 mL optical 96-well semi-sk":E2
"MicroAmp™ 0.2 mL Fast Optical 96-well se":E3	20	Sample	Reagent	1	a.u.			"MicroAmp™ 0.2 mL Fast Optical 96-well se":E3	7	"MicroAmp™ 0.3 mL optical 96-well semi-sk":E3
"MicroAmp™ 0.2 mL Fast Optical 96-well se":E4	20	Sample	Reagent	1	a.u.			"MicroAmp™ 0.2 mL Fast Optical 96-well se":E4	7	"MicroAmp™ 0.3 mL optical 96-well semi-sk":E4
"MicroAmp™ 0.2 mL Fast Optical 96-well se":E5	20	Sample	Reagent	1	a.u.			"MicroAmp™ 0.2 mL Fast Optical 96-well se":E5	7	"MicroAmp™ 0.3 mL optical 96-well semi-sk":E5
"MicroAmp™ 0.2 mL Fast Optical 96-well se":E6	20	Sample	Reagent	1	a.u.			"MicroAmp™ 0.2 mL Fast Optical 96-well se":E6	7	"MicroAmp™ 0.3 mL optical 96-well semi-sk":E6
"MicroAmp™ 0.2 mL Fast Optical 96-well se":E7	20	Sample	Reagent	1	a.u.			"MicroAmp™ 0.2 mL Fast Optical 96-well se":E7	7	"MicroAmp™ 0.3 mL optical 96-well semi-sk":E7
"MicroAmp™ 0.2 mL Fast Optical 96-well se":E8	20	Sample	Reagent	1	a.u.			"MicroAmp™ 0.2 mL Fast Optical 96-well se":E8	7	"MicroAmp™ 0.3 mL optical 96-well semi-sk":E8
"MicroAmp™ 0.2 mL Fast Optical 96-well se":E9	20	Sample	Reagent	1	a.u.			"MicroAmp™ 0.2 mL Fast Optical 96-well se":E9	7	"MicroAmp™ 0.3 mL optical 96-well semi-sk":E9
"MicroAmp™ 0.2 mL Fast Optical 96-well se":E10	20	Sample	Reagent	1	a.u.			"MicroAmp™ 0.2 mL Fast Optical 96-well se":E10	7	"MicroAmp™ 0.3 mL optical 96-well semi-sk":E10
"MicroAmp™ 0.2 mL Fast Optical 96-well se":E11	20	Sample	Reagent	1	a.u.			"MicroAmp™ 0.2 mL Fast Optical 96-well se":E11	7	"MicroAmp™ 0.3 mL optical 96-well semi-sk":E11
"MicroAmp™ 0.2 mL Fast Optical 96-well se":E12	20	Sample	Reagent	1	a.u.			"MicroAmp™ 0.2 mL Fast Optical 96-well se":E12	7	"MicroAmp™ 0.3 mL optical 96-well semi-sk":E12
"MicroAmp™ 0.2 mL Fast Optical 96-well se":F1	20	Sample	Reagent	1	a.u.			"MicroAmp™ 0.2 mL Fast Optical 96-well se":F1	7	"MicroAmp™ 0.3 mL optical 96-well semi-sk":F1
"MicroAmp™ 0.2 mL Fast Optical 96-well se":F2	20	Sample	Reagent	1	a.u.			"MicroAmp™ 0.2 mL Fast Optical 96-well se":F2	7	"MicroAmp™ 0.3 mL optical 96-well semi-sk":F2
"MicroAmp™ 0.2 mL Fast Optical 96-well se":F3	20	Sample	Reagent	1	a.u.			"MicroAmp™ 0.2 mL Fast Optical 96-well se":F3	7	"MicroAmp™ 0.3 mL optical 96-well semi-sk":F3
"MicroAmp™ 0.2 mL Fast Optical 96-well se":F4	20	Sample	Reagent	1	a.u.			"MicroAmp™ 0.2 mL Fast Optical 96-well se":F4	7	"MicroAmp™ 0.3 mL optical 96-well semi-sk":F4
"MicroAmp™ 0.2 mL Fast Optical 96-well se":F5	20	Sample	Reagent	1	a.u.			"MicroAmp™ 0.2 mL Fast Optical 96-well se":F5	7	"MicroAmp™ 0.3 mL optical 96-well semi-sk":F5
"MicroAmp™ 0.2 mL Fast Optical 96-well se":F6	20	Sample	Reagent	1	a.u.			"MicroAmp™ 0.2 mL Fast Optical 96-well se":F6	7	"MicroAmp™ 0.3 mL optical 96-well semi-sk":F6
"MicroAmp™ 0.2 mL Fast Optical 96-well se":F7	20	Sample	Reagent	1	a.u.			"MicroAmp™ 0.2 mL Fast Optical 96-well se":F7	7	"MicroAmp™ 0.3 mL optical 96-well semi-sk":F7
"MicroAmp™ 0.2 mL Fast Optical 96-well se":F8	20	Sample	Reagent	1	a.u.			"MicroAmp™ 0.2 mL Fast Optical 96-well se":F8	7	"MicroAmp™ 0.3 mL optical 96-well semi-sk":F8
"MicroAmp™ 0.2 mL Fast Optical 96-well se":F9	20	Sample	Reagent	1	a.u.			"MicroAmp™ 0.2 mL Fast Optical 96-well se":F9	7	"MicroAmp™ 0.3 mL optical 96-well semi-sk":F9
"MicroAmp™ 0.2 mL Fast Optical 96-well se":F10	20	Sample	Reagent	1	a.u.			"MicroAmp™ 0.2 mL Fast Optical 96-well se":F10	7	"MicroAmp™ 0.3 mL optical 96-well semi-sk":F10
"MicroAmp™ 0.2 mL Fast Optical 96-well se":F11	20	Sample	Reagent	1	a.u.			"MicroAmp™ 0.2 mL Fast Optical 96-well se":F11	7	"MicroAmp™ 0.3 mL optical 96-well semi-sk":F11
"MicroAmp™ 0.2 mL Fast Optical 96-well se":F12	20	Sample	Reagent	1	a.u.			"MicroAmp™ 0.2 mL Fast Optical 96-well se":F12	7	"MicroAmp™ 0.3 mL optical 96-well semi-sk":F12
"MicroAmp™ 0.2 mL Fast Optical 96-well se":G1	20	Sample	Reagent	1	a.u.			"MicroAmp™ 0.2 mL Fast Optical 96-well se":G1	7	"MicroAmp™ 0.3 mL optical 96-well semi-sk":G1
"MicroAmp™ 0.2 mL Fast Optical 96-well se":G2	20	Sample	Reagent	1	a.u.			"MicroAmp™ 0.2 mL Fast Optical 96-well se":G2	7	"MicroAmp™ 0.3 mL optical 96-well semi-sk":G2
"MicroAmp™ 0.2 mL Fast Optical 96-well se":G3	20	Sample	Reagent	1	a.u.			"MicroAmp™ 0.2 mL Fast Optical 96-well se":G3	7	"MicroAmp™ 0.3 mL optical 96-well semi-sk":G3
"MicroAmp™ 0.2 mL Fast Optical 96-well se":G4	20	Sample	Reagent	1	a.u.			"MicroAmp™ 0.2 mL Fast Optical 96-well se":G4	7	"MicroAmp™ 0.3 mL optical 96-well semi-sk":G4
"MicroAmp™ 0.2 mL Fast Optical 96-well se":G5	20	Sample	Reagent	1	a.u.			"MicroAmp™ 0.2 mL Fast Optical 96-well se":G5	7	"MicroAmp™ 0.3 mL optical 96-well semi-sk":G5
"MicroAmp™ 0.2 mL Fast Optical 96-well se":G6	20	Sample	Reagent	1	a.u.			"MicroAmp™ 0.2 mL Fast Optical 96-well se":G6	7	"MicroAmp™ 0.3 mL optical 96-well semi-sk":G6
"MicroAmp™ 0.2 mL Fast Optical 96-well se":G7	20	Sample	Reagent	1	a.u.			"MicroAmp™ 0.2 mL Fast Optical 96-well se":G7	7	"MicroAmp™ 0.3 mL optical 96-well semi-sk":G7
"MicroAmp™ 0.2 mL Fast Optical 96-well se":G8	20	Sample	Reagent	1	a.u.			"MicroAmp™ 0.2 mL Fast Optical 96-well se":G8	7	"MicroAmp™ 0.3 mL optical 96-well semi-sk":G8
"MicroAmp™ 0.2 mL Fast Optical 96-well se":G9	20	Sample	Reagent	1	a.u.			"MicroAmp™ 0.2 mL Fast Optical 96-well se":G9	7	"MicroAmp™ 0.3 mL optical 96-well semi-sk":G9
"MicroAmp™ 0.2 mL Fast Optical 96-well se":G10	20	Sample	Reagent	1	a.u.			"MicroAmp™ 0.2 mL Fast Optical 96-well se":G10	7	"MicroAmp™ 0.3 mL optical 96-well semi-sk":G10
"MicroAmp™ 0.2 mL Fast Optical 96-well se":G11	20	Sample	Reagent	1	a.u.			"MicroAmp™ 0.2 mL Fast Optical 96-well se":G11	7	"MicroAmp™ 0.3 mL optical 96-well semi-sk":G11
"MicroAmp™ 0.2 mL Fast Optical 96-well se":G12	20	Sample	Reagent	1	a.u.			"MicroAmp™ 0.2 mL Fast Optical 96-well se":G12	7	"MicroAmp™ 0.3 mL optical 96-well semi-sk":G12
"MicroAmp™ 0.2 mL Fast Optical 96-well se":H1	20	Sample	Reagent	1	a.u.			"MicroAmp™ 0.2 mL Fast Optical 96-well se":H1	7	"MicroAmp™ 0.3 mL optical 96-well semi-sk":H1
"MicroAmp™ 0.2 mL Fast Optical 96-well se":H2	20	Sample	Reagent	1	a.u.			"MicroAmp™ 0.2 mL Fast Optical 96-well se":H2	7	"MicroAmp™ 0.3 mL optical 96-well semi-sk":H2
"MicroAmp™ 0.2 mL Fast Optical 96-well se":H3	20	Sample	Reagent	1	a.u.			"MicroAmp™ 0.2 mL Fast Optical 96-well se":H3	7	"MicroAmp™ 0.3 mL optical 96-well semi-sk":H3
"MicroAmp™ 0.2 mL Fast Optical 96-well se":H4	20	Sample	Reagent	1	a.u.			"MicroAmp™ 0.2 mL Fast Optical 96-well se":H4	7	"MicroAmp™ 0.3 mL optical 96-well semi-sk":H4
"MicroAmp™ 0.2 mL Fast Optical 96-well se":H5	20	Sample	Reagent	1	a.u.			"MicroAmp™ 0.2 mL Fast Optical 96-well se":H5	7	"MicroAmp™ 0.3 mL optical 96-well semi-sk":H5
"MicroAmp™ 0.2 mL Fast Optical 96-well se":H6	20	Sample	Reagent	1	a.u.			"MicroAmp™ 0.2 mL Fast Optical 96-well se":H6	7	"MicroAmp™ 0.3 mL optical 96-well semi-sk":H6
"MicroAmp™ 0.2 mL Fast Optical 96-well se":H7	20	Sample	Reagent	1	a.u.			"MicroAmp™ 0.2 mL Fast Optical 96-well se":H7	7	"MicroAmp™ 0.3 mL optical 96-well semi-sk":H7
"MicroAmp™ 0.2 mL Fast Optical 96-well se":H8	20	Sample	Reagent	1	a.u.			"MicroAmp™ 0.2 mL Fast Optical 96-well se":H8	7	"MicroAmp™ 0.3 mL optical 96-well semi-sk":H8
"MicroAmp™ 0.2 mL Fast Optical 96-well se":H9	20	Sample	Reagent	1	a.u.			"MicroAmp™ 0.2 mL Fast Optical 96-well se":H9	7	"MicroAmp™ 0.3 mL optical 96-well semi-sk":H9
"MicroAmp™ 0.2 mL Fast Optical 96-well se":H10	20	Sample	Reagent	1	a.u.			"MicroAmp™ 0.2 mL Fast Optical 96-well se":H10	7	"MicroAmp™ 0.3 mL optical 96-well semi-sk":H10
"MicroAmp™ 0.2 mL Fast Optical 96-well se":H11	20	Sample	Reagent	1	a.u.			"MicroAmp™ 0.2 mL Fast Optical 96-well se":H11	7	"MicroAmp™ 0.3 mL optical 96-well semi-sk":H11
"MicroAmp™ 0.2 mL Fast Optical 96-well se":H12	20	Sample	Reagent	1	a.u.			"MicroAmp™ 0.2 mL Fast Optical 96-well se":H12	7	"MicroAmp™ 0.3 mL optical 96-well semi-sk":H12
"MicroAmp™ 0.3 mL optical 96-well semi-sk":A1	100	01 Yellow Buffer	Reagent	1	a.u.					
"MicroAmp™ 0.3 mL optical 96-well semi-sk":A2	100	01 Yellow Buffer	Reagent	1	a.u.					
"MicroAmp™ 0.3 mL optical 96-well semi-sk":A3	100	01 Yellow Buffer	Reagent	1	a.u.					
"MicroAmp™ 0.3 mL optical 96-well semi-sk":A4	100	01 Yellow Buffer	Reagent	1	a.u.					
"MicroAmp™ 0.3 mL optical 96-well semi-sk":A5	100	01 Yellow Buffer	Reagent	1	a.u.					
"MicroAmp™ 0.3 mL optical 96-well semi-sk":A6	100	01 Yellow Buffer	Reagent	1	a.u.					
"MicroAmp™ 0.3 mL optical 96-well semi-sk":A7	100	01 Yellow Buffer	Reagent	1	a.u.					
"MicroAmp™ 0.3 mL optical 96-well semi-sk":A8	100	01 Yellow Buffer	Reagent	1	a.u.					
"MicroAmp™ 0.3 mL optical 96-well semi-sk":A9	100	01 Yellow Buffer	Reagent	1	a.u.					
"MicroAmp™ 0.3 mL optical 96-well semi-sk":A10	100	01 Yellow Buffer	Reagent	1	a.u.					
"MicroAmp™ 0.3 mL optical 96-well semi-sk":A11	100	01 Yellow Buffer	Reagent	1	a.u.					
"MicroAmp™ 0.3 mL optical 96-well semi-sk":A12	100	01 Yellow Buffer	Reagent	1	a.u.					
"MicroAmp™ 0.3 mL optical 96-well semi-sk":B1	100	01 Yellow Buffer	Reagent	1	a.u.					
"MicroAmp™ 0.3 mL optical 96-well semi-sk":B2	100	01 Yellow Buffer	Reagent	1	a.u.					
"MicroAmp™ 0.3 mL optical 96-well semi-sk":B3	100	01 Yellow Buffer	Reagent	1	a.u.					
"MicroAmp™ 0.3 mL optical 96-well semi-sk":B4	100	01 Yellow Buffer	Reagent	1	a.u.					
"MicroAmp™ 0.3 mL optical 96-well semi-sk":B5	100	01 Yellow Buffer	Reagent	1	a.u.					
"MicroAmp™ 0.3 mL optical 96-well semi-sk":B6	100	01 Yellow Buffer	Reagent	1	a.u.					
"MicroAmp™ 0.3 mL optical 96-well semi-sk":B7	100	01 Yellow Buffer	Reagent	1	a.u.					
"MicroAmp™ 0.3 mL optical 96-well semi-sk":B8	100	01 Yellow Buffer	Reagent	1	a.u.					
"MicroAmp™ 0.3 mL optical 96-well semi-sk":B9	100	01 Yellow Buffer	Reagent	1	a.u.					
"MicroAmp™ 0.3 mL optical 96-well semi-sk":B10	100	01 Yellow Buffer	Reagent	1	a.u.					
"MicroAmp™ 0.3 mL optical 96-well semi-sk":B11	100	01 Yellow Buffer	Reagent	1	a.u.					
"MicroAmp™ 0.3 mL optical 96-well semi-sk":B12	100	01 Yellow Buffer	Reagent	1	a.u.					
"MicroAmp™ 0.3 mL optical 96-well semi-sk":C1	100	01 Yellow Buffer	Reagent	1	a.u.					
"MicroAmp™ 0.3 mL optical 96-well semi-sk":C2	100	01 Yellow Buffer	Reagent	1	a.u.					
"MicroAmp™ 0.3 mL optical 96-well semi-sk":C3	100	01 Yellow Buffer	Reagent	1	a.u.					
"MicroAmp™ 0.3 mL optical 96-well semi-sk":C4	100	01 Yellow Buffer	Reagent	1	a.u.					
"MicroAmp™ 0.3 mL optical 96-well semi-sk":C5	100	01 Yellow Buffer	Reagent	1	a.u.					
"MicroAmp™ 0.3 mL optical 96-well semi-sk":C6	100	01 Yellow Buffer	Reagent	1	a.u.					
"MicroAmp™ 0.3 mL optical 96-well semi-sk":C7	100	01 Yellow Buffer	Reagent	1	a.u.					
"MicroAmp™ 0.3 mL optical 96-well semi-sk":C8	100	01 Yellow Buffer	Reagent	1	a.u.					
"MicroAmp™ 0.3 mL optical 96-well semi-sk":C9	100	01 Yellow Buffer	Reagent	1	a.u.					
"MicroAmp™ 0.3 mL optical 96-well semi-sk":C10	100	01 Yellow Buffer	Reagent	1	a.u.					
"MicroAmp™ 0.3 mL optical 96-well semi-sk":C11	100	01 Yellow Buffer	Reagent	1	a.u.					
"MicroAmp™ 0.3 mL optical 96-well semi-sk":C12	100	01 Yellow Buffer	Reagent	1	a.u.					
"MicroAmp™ 0.3 mL optical 96-well semi-sk":D1	100	01 Yellow Buffer	Reagent	1	a.u.					
"MicroAmp™ 0.3 mL optical 96-well semi-sk":D2	100	01 Yellow Buffer	Reagent	1	a.u.					
"MicroAmp™ 0.3 mL optical 96-well semi-sk":D3	100	01 Yellow Buffer	Reagent	1	a.u.					
"MicroAmp™ 0.3 mL optical 96-well semi-sk":D4	100	01 Yellow Buffer	Reagent	1	a.u.					
"MicroAmp™ 0.3 mL optical 96-well semi-sk":D5	100	01 Yellow Buffer	Reagent	1	a.u.					
"MicroAmp™ 0.3 mL optical 96-well semi-sk":D6	100	01 Yellow Buffer	Reagent	1	a.u.					
"MicroAmp™ 0.3 mL optical 96-well semi-sk":D7	100	01 Yellow Buffer	Reagent	1	a.u.					
"MicroAmp™ 0.3 mL optical 96-well semi-sk":D8	100	01 Yellow Buffer	Reagent	1	a.u.					
"MicroAmp™ 0.3 mL optical 96-well semi-sk":D9	100	01 Yellow Buffer	Reagent	1	a.u.					
"MicroAmp™ 0.3 mL optical 96-well semi-sk":D10	100	01 Yellow Buffer	Reagent	1	a.u.					
"MicroAmp™ 0.3 mL optical 96-well semi-sk":D11	100	01 Yellow Buffer	Reagent	1	a.u.					
"MicroAmp™ 0.3 mL optical 96-well semi-sk":D12	100	01 Yellow Buffer	Reagent	1	a.u.					
"MicroAmp™ 0.3 mL optical 96-well semi-sk":E1	100	01 Yellow Buffer	Reagent	1	a.u.					
"MicroAmp™ 0.3 mL optical 96-well semi-sk":E2	100	01 Yellow Buffer	Reagent	1	a.u.					
"MicroAmp™ 0.3 mL optical 96-well semi-sk":E3	100	01 Yellow Buffer	Reagent	1	a.u.					
"MicroAmp™ 0.3 mL optical 96-well semi-sk":E4	100	01 Yellow Buffer	Reagent	1	a.u.					
"MicroAmp™ 0.3 mL optical 96-well semi-sk":E5	100	01 Yellow Buffer	Reagent	1	a.u.					
"MicroAmp™ 0.3 mL optical 96-well semi-sk":E6	100	01 Yellow Buffer	Reagent	1	a.u.					
"MicroAmp™ 0.3 mL optical 96-well semi-sk":E7	100	01 Yellow Buffer	Reagent	1	a.u.					
"MicroAmp™ 0.3 mL optical 96-well semi-sk":E8	100	01 Yellow Buffer	Reagent	1	a.u.					
"MicroAmp™ 0.3 mL optical 96-well semi-sk":E9	100	01 Yellow Buffer	Reagent	1	a.u.					
"MicroAmp™ 0.3 mL optical 96-well semi-sk":E10	100	01 Yellow Buffer	Reagent	1	a.u.					
"MicroAmp™ 0.3 mL optical 96-well semi-sk":E11	100	01 Yellow Buffer	Reagent	1	a.u.					
"MicroAmp™ 0.3 mL optical 96-well semi-sk":E12	100	01 Yellow Buffer	Reagent	1	a.u.					
"MicroAmp™ 0.3 mL optical 96-well semi-sk":F1	100	01 Yellow Buffer	Reagent	1	a.u.					
"MicroAmp™ 0.3 mL optical 96-well semi-sk":F2	100	01 Yellow Buffer	Reagent	1	a.u.					
"MicroAmp™ 0.3 mL optical 96-well semi-sk":F3	100	01 Yellow Buffer	Reagent	1	a.u.					
"MicroAmp™ 0.3 mL optical 96-well semi-sk":F4	100	01 Yellow Buffer	Reagent	1	a.u.					
"MicroAmp™ 0.3 mL optical 96-well semi-sk":F5	100	01 Yellow Buffer	Reagent	1	a.u.					
"MicroAmp™ 0.3 mL optical 96-well semi-sk":F6	100	01 Yellow Buffer	Reagent	1	a.u.					
"MicroAmp™ 0.3 mL optical 96-well semi-sk":F7	100	01 Yellow Buffer	Reagent	1	a.u.					
"MicroAmp™ 0.3 mL optical 96-well semi-sk":F8	100	01 Yellow Buffer	Reagent	1	a.u.					
"MicroAmp™ 0.3 mL optical 96-well semi-sk":F9	100	01 Yellow Buffer	Reagent	1	a.u.					
"MicroAmp™ 0.3 mL optical 96-well semi-sk":F10	100	01 Yellow Buffer	Reagent	1	a.u.					
"MicroAmp™ 0.3 mL optical 96-well semi-sk":F11	100	01 Yellow Buffer	Reagent	1	a.u.					
"MicroAmp™ 0.3 mL optical 96-well semi-sk":F12	100	01 Yellow Buffer	Reagent	1	a.u.					
"MicroAmp™ 0.3 mL optical 96-well semi-sk":G1	100	01 Yellow Buffer	Reagent	1	a.u.					
"MicroAmp™ 0.3 mL optical 96-well semi-sk":G2	100	01 Yellow Buffer	Reagent	1	a.u.					
"MicroAmp™ 0.3 mL optical 96-well semi-sk":G3	100	01 Yellow Buffer	Reagent	1	a.u.					
"MicroAmp™ 0.3 mL optical 96-well semi-sk":G4	100	01 Yellow Buffer	Reagent	1	a.u.					
"MicroAmp™ 0.3 mL optical 96-well semi-sk":G5	100	01 Yellow Buffer	Reagent	1	a.u.					
"MicroAmp™ 0.3 mL optical 96-well semi-sk":G6	100	01 Yellow Buffer	Reagent	1	a.u.					
"MicroAmp™ 0.3 mL optical 96-well semi-sk":G7	100	01 Yellow Buffer	Reagent	1	a.u.					
"MicroAmp™ 0.3 mL optical 96-well semi-sk":G8	100	01 Yellow Buffer	Reagent	1	a.u.					
"MicroAmp™ 0.3 mL optical 96-well semi-sk":G9	100	01 Yellow Buffer	Reagent	1	a.u.					
"MicroAmp™ 0.3 mL optical 96-well semi-sk":G10	100	01 Yellow Buffer	Reagent	1	a.u.					
"MicroAmp™ 0.3 mL optical 96-well semi-sk":G11	100	01 Yellow Buffer	Reagent	1	a.u.					
"MicroAmp™ 0.3 mL optical 96-well semi-sk":G12	100	01 Yellow Buffer	Reagent	1	a.u.					
"MicroAmp™ 0.3 mL optical 96-well semi-sk":H1	100	01 Yellow Buffer	Reagent	1	a.u.					
"MicroAmp™ 0.3 mL optical 96-well semi-sk":H2	100	01 Yellow Buffer	Reagent	1	a.u.					
"MicroAmp™ 0.3 mL optical 96-well semi-sk":H3	100	01 Yellow Buffer	Reagent	1	a.u.					
"MicroAmp™ 0.3 mL optical 96-well semi-sk":H4	100	01 Yellow Buffer	Reagent	1	a.u.					
"MicroAmp™ 0.3 mL optical 96-well semi-sk":H5	100	01 Yellow Buffer	Reagent	1	a.u.					
"MicroAmp™ 0.3 mL optical 96-well semi-sk":H6	100	01 Yellow Buffer	Reagent	1	a.u.					
"MicroAmp™ 0.3 mL optical 96-well semi-sk":H7	100	01 Yellow Buffer	Reagent	1	a.u.					
"MicroAmp™ 0.3 mL optical 96-well semi-sk":H8	100	01 Yellow Buffer	Reagent	1	a.u.					
"MicroAmp™ 0.3 mL optical 96-well semi-sk":H9	100	01 Yellow Buffer	Reagent	1	a.u.					
"MicroAmp™ 0.3 mL optical 96-well semi-sk":H10	100	01 Yellow Buffer	Reagent	1	a.u.					
"MicroAmp™ 0.3 mL optical 96-well semi-sk":H11	100	01 Yellow Buffer	Reagent	1	a.u.					
"MicroAmp™ 0.3 mL optical 96-well semi-sk":H12	100	01 Yellow Buffer	Reagent	1	a.u.					
